# Supplementary material for: Who is most at risk of dying if infected with SARS-CoV-2? A mortality risk factor analysis using machine learning of patients with COVID-19 over time: a large population-based cohort study in Mexico
Source: BMJ Open. 2023 Sep 22;13(9):e072436. doi: 10.1136/bmjopen-2023-072436 (PMC10533798; doi:10.1136/bmjopen-2023-072436)
Supplement: Supplementary data [file bmjopen-2023-072436supp001.pdf]

Supplemental Material

Who is most at risk of dying if infected with SARS-CoV-2? A mortality risk factor analysis using machine learning of COVID-19 patients over time: a large population-based cohort study in Mexico.

Lauren D. Liao,* Alan E. Hubbard, Juan Pablo Gutiérrez, Arturo Juárez-Flores, Kendall Kikkawa, Ronit Gupta, Yana Yarmolich, Iván de Jesús Ascencio-Montiel, Stefano M. Bertozzi

Liao LD, *et al. BMJ Open* 2023; 13:e072436. doi: 10.1136/bmjopen-2023-072436

Table of Contents

Table S1. Complete table of baseline variables and pre-existing conditions 3

Table S2. Weighted combination of the super learner fit 7

Table S3. Top 5 ranked most important variables for prediction 8

Table S4. Targeted maximum likelihood estimation adjusted mortality risk, with or without the pre-existing condition 9

Fig. S1. Flowchart for analytic sample development 10

Fig. S2. Age distribution for laboratory-confirmed COVID-19 patients 11

Fig. S3. Prevalence of pre-existing conditions prevalence over time 12

Fig. S4. Prediction variable importance predicted using the super learner fit 13

Fig. S5. Relative risk for each pre-existing condition associated with mortality 14

Table S1. Complete table of baseline variables and pre-existing conditions

	All time (2020/03-2021/11)	Phase 1 (2020/03-2020/10)	Phase 2 (2020/11-2021/03)	Phase 3 (2021/04-2021/11)
sample size	1,423,720	303,278	425,698	694,744
Demographic variables				
Age in years (mean (SD))	42.15 (15.70)	46.41 (16.04)	44.89 (16.27)	38.61 (14.34)
Sex = male (%)	729,782 (51.3)	158,248 (52.2)	218,165 (51.2)	353,369 (50.9)
Insured by IMSS = 1(%)	1,358,440 (95.4)	288,588 (95.2)	402,754 (94.6)	667,098 (96.0)
Indigenous = 1 (%)	7,381 (0.5)	2,200 (0.7)	1,628 (0.4)	3,553 (0.5)
Year-month patient initiated care				
2020/03	1,061 (0.1)	1,061 (0.3)	0 (0.0)	0 (0.0)
2020/04	10,832 (0.8)	10,832 (3.6)	0 (0.0)	0 (0.0)
2020/05	30,720 (2.2)	30,720 (10.1)	0 (0.0)	0 (0.0)
2020/06	51,079 (3.6)	51,079 (16.8)	0 (0.0)	0 (0.0)
2020/07	60,780 (4.3)	60,780 (20.0)	0 (0.0)	0 (0.0)
2020/08	49,618 (3.5)	49,618 (16.4)	0 (0.0)	0 (0.0)
2020/09	44,758 (3.1)	44,758 (14.8)	0 (0.0)	0 (0.0)
2020/10	54,430 (3.8)	54,430 (17.9)	0 (0.0)	0 (0.0)
2020/11	65,437 (4.6)	0 (0.0)	65,437 (15.4)	0 (0.0)
2020/12	93,748 (6.6)	0 (0.0)	93,748 (22.0)	0 (0.0)
2021/01	145,858 (10.2)	0 (0.0)	145,858 (34.3)	0 (0.0)
2021/02	68,421 (4.8)	0 (0.0)	68,421 (16.1)	0 (0.0)
2021/03	52,234 (3.7)	0 (0.0)	52,234 (12.3)	0 (0.0)
2021/04	35,181 (2.5)	0 (0.0)	0 (0.0)	35,181 (5.1)

Liao LD, *et al. BMJ Open* 2023; 13:e072436. doi: 10.1136/bmjopen-2023-072436

2021/05	26,300 (1.8)	0 (0.0)	0 (0.0)	26,300 (3.8)
2021/06	45,986 (3.2)	0 (0.0)	0 (0.0)	45,986 (6.6)
2021/07	170,212 (12.0)	0 (0.0)	0 (0.0)	170,212 (24.5)
2021/08	249,477 (17.5)	0 (0.0)	0 (0.0)	249,477 (35.9)
2021/09	116,569 (8.2)	0 (0.0)	0 (0.0)	116,569 (16.8)
2021/10	48,515 (3.4)	0 (0.0)	0 (0.0)	48,515 (7.0)
2021/11	2,504 (0.2)	0 (0.0)	0 (0.0)	2,504 (0.4)
Mexican states (%)				
Aguascalientes	26,420 (1.9)	6,897 (2.3)	12,350 (2.9)	7,173 (1.0)
Baja California	43,925 (3.1)	13,677 (4.5)	14,188 (3.3)	16,060 (2.3)
Baja California Sur	24,521 (1.7)	4,300 (1.4)	5,423 (1.3)	14,798 (2.1)
Campeche	9,557 (0.7)	1,728 (0.6)	765 (0.2)	7,064 (1.0)
CDMX 1 Noroeste	32,552 (2.3)	5,374 (1.8)	13,174 (3.1)	14,004 (2.0)
CDMX 2 Noreste	54,249 (3.8)	11,370 (3.7)	20,273 (4.8)	22,606 (3.3)
CDMX 3 Suroeste	42,896 (3.0)	9,701 (3.2)	17,588 (4.1)	15,607 (2.2)
CDMX 4 Sureste	62,097 (4.4)	13,248 (4.4)	25,899 (6.1)	22,950 (3.3)
Chiapas	14,826 (1.0)	2,836 (0.9)	1,801 (0.4)	10,189 (1.5)
Chihuahua	23,229 (1.6)	6,489 (2.1)	6,879 (1.6)	9,861 (1.4)
Coahuila	48,933 (3.4)	16,355 (5.4)	15,459 (3.6)	17,119 (2.5)
Colima	18,310 (1.3)	2,997 (1.0)	2,633 (0.6)	12,680 (1.8)
Durango	19,738 (1.4)	6,228 (2.1)	6,674 (1.6)	6,836 (1.0)
Guanajuato	61,570 (4.3)	11,595 (3.8)	29,274 (6.9)	20,701 (3.0)
Guerrero	23,871 (1.7)	4,502 (1.5)	3,920 (0.9)	15,449 (2.2)
Hidalgo	23,673 (1.7)	4,658 (1.5)	8,266 (1.9)	10,749 (1.5)
Jalisco	104,054 (7.3)	19,491 (6.4)	27,868 (6.5)	56,695 (8.2)

Mexico Oriente	108,067 (7.6)	20,368 (6.7)	39,633 (9.3)	48,066 (6.9)
Mexico Poniente	48,973 (3.4)	11,632 (3.8)	17,026 (4.0)	20,315 (2.9)
Michoacan	30,570 (2.1)	6,246 (2.1)	7,221 (1.7)	17,103 (2.5)
Morelos	18,797 (1.3)	2,845 (0.9)	6,817 (1.6)	9,135 (1.3)
Nayarit	23,934 (1.7)	3,378 (1.1)	2,994 (0.7)	17,562 (2.5)
Nuevo Leon	105,912 (7.4)	23,776 (7.8)	30,114 (7.1)	52,022 (7.5)
Oaxaca	24,324 (1.7)	4,493 (1.5)	5,391 (1.3)	14,440 (2.1)
Puebla	50,998 (3.6)	9,287 (3.1)	17,932 (4.2)	23,779 (3.4)
Queretaro	41,977 (2.9)	4,707 (1.6)	19,259 (4.5)	18,011 (2.6)
Quintana Roo	38,390 (2.7)	4,607 (1.5)	4,542 (1.1)	29,241 (4.2)
San Luis Potosi	26,118 (1.8)	7,353 (2.4)	7,573 (1.8)	11,192 (1.6)
Sinaloa	44,333 (3.1)	11,030 (3.6)	9,405 (2.2)	23,898 (3.4)
Sonora	27,691 (1.9)	7,245 (2.4)	6,083 (1.4)	14,363 (2.1)
Tabasco	16,004 (1.1)	2,622 (0.9)	1,719 (0.4)	11,663 (1.7)
Tamaulipas	38,941 (2.7)	8,504 (2.8)	7,442 (1.7)	22,995 (3.3)
Tlaxcala	13,809 (1.0)	2,769 (0.9)	4,957 (1.2)	6,083 (0.9)
Veracruz Norte	41,804 (2.9)	10,002 (3.3)	6,801 (1.6)	25,001 (3.6)
Veracruz Sur	35,019 (2.5)	10,292 (3.4)	4,970 (1.2)	19,757 (2.8)
Yucatan	35,126 (2.5)	5,968 (2.0)	5,091 (1.2)	24,067 (3.5)
Zacatecas	18,512 (1.3)	4,708 (1.6)	8,294 (1.9)	5,510 (0.8)
Pre-existing conditions				
Asthma = yes (%)	25,297 (1.8)	7,951 (2.6)	7,765 (1.8)	9,581 (1.4)
Cardiovascular disease = yes (%)	17,816 (1.3)	6,643 (2.2)	6,389 (1.5)	4,784 (0.7)
Chronic liver disease = yes (%)	1,875 (0.1)	710 (0.2)	668 (0.2)	497 (0.1)
COPD = yes (%)	15,390 (1.1)	5,825 (1.9)	5,496 (1.3)	4,069 (0.6)

Liao LD, *et al. BMJ Open* 2023; 13:e072436. doi: 10.1136/bmjopen-2023-072436

Diabetes = yes (%)	169,869 (11.9)	55,551 (18.3)	61,120 (14.4)	53,198 (7.7)
Hemolytic anemia = yes (%)	705 (0.0)	276 (0.1)	246 (0.1)	183 (0.0)
HIV = yes (%)	4,717 (0.3)	1,133 (0.4)	1,425 (0.3)	2,159 (0.3)
Hypertension = yes (%)	228,901 (16.1)	72,615 (23.9)	83,735 (19.7)	72,551 (10.4)
Immunosuppression = yes (%)	10,434 (0.7)	4,102 (1.4)	3,453 (0.8)	2,879 (0.4)
Neurological disease = yes (%)	1,645 (0.1)	544 (0.2)	559 (0.1)	542 (0.1)
Obesity = yes (%)	181,736 (12.8)	55,965 (18.5)	60,217 (14.1)	65,554 (9.4)
Smoking = yes (%)	87,161 (6.1)	21,253 (7.0)	28,346 (6.7)	37,562 (5.4)
Cancer diagnosis = yes (%)	3,751 (0.3)	1,178 (0.4)	1,317 (0.3)	1,256 (0.2)
Renal disease diagnosis = yes (%)	24,099 (1.7)	8,912 (2.9)	8,555 (2.0)	6,632 (1.0)
Tuberculosis = yes (%)	675 (0.0)	203 (0.1)	218 (0.1)	254 (0.0)

CDMX: Ciudad de México; COPD: Chronic obstructive pulmonary disease; HIV: human immunodeficiency virus; SD: standard deviation
Mexican states refer to where the patient was treated

Liao LD, *et al. BMJ Open* 2023; 13:e072436. doi: 10.1136/bmjopen-2023-072436

Table S2. Weighted combination of the super learner fit

Machine learning candidate algorithm	Weights	Mean squared error	Standard error
Bayesian additive regression trees	0	0.266	0.0002
Bayesian generalized linear model	0	0.067	0.0003
Elastic net regression	0	0.068	0.0004
Empirical mean	0	0.094	0.0005
XGBoost (multiple tuning)	0.596 (combined)	0.065 (on average)	0.0003 (on average)
Generalized additive model	0.222	0.066	0.0004
LASSO regression	0	0.067	0.0004
Logistic regression	0	0.067	0.0004
Multivariate Adaptive Regression Splines	0	0.068	0.0004
Random forest	0.181	0.066	0.0002
Ridge regression	0	0.067	0.0002

LASSO: least absolute shrinkage and selection operator; XGBoost: extreme gradient boosting
XGBoost coefficients are combined; mean squared error and standard error were averaged.

Liao LD, *et al. BMJ Open* 2023; 13:e072436. doi: 10.1136/bmjopen-2023-072436

Table S3. Top 5 ranked most important variables for prediction

	All time (2020/03-2021/11)	Phase 1 (2020/03-2020/10)	Phase 2 (2020/11-2021/03)	Phase 3 (2021/04-2021/11)
Rank 1	Age 0.147	Age 0.209	Age 0.208	Age 0.069
Rank 2	Year-month patient initiated care 0.014	Renal disease 0.008	Mexican state 0.007	Renal disease 0.004
Rank 3	Renal disease 0.005	Sex 0.007	Renal disease 0.006	Mexican state 0.003
Rank 4	Mexican state 0.004	Year-month patient initiated care 0.007	Insured by IMSS 0.006	Diabetes 0.003
Rank 5	Sex 0.004	Mexican state 0.006	Sex 0.006	Insured by IMSS 0.002

IMSS: Mexican Institute of Social Security
Mexican state refers to where the patient was treated.
Measured by the log-likelihood difference in prediction pre-post permutation of each variable while holding all others constant.

Liao LD, *et al. BMJ Open* 2023; 13:e072436. doi: 10.1136/bmjopen-2023-072436

Table S4. Targeted maximum likelihood estimation adjusted mortality risk, with or without the pre-existing condition

	All time (2020/03-2021/11)		Phase 1 (2020/03-2020/10)		Phase 2 (2020/11-2021/03)		Phase 3 (2021/04-2021/11)	
	with	without	with	without	with	without	with	without
Renal disease	0.381	0.101	0.439	0.170	0.425	0.142	0.305	0.046
Diabetes	0.173	0.094	0.247	0.161	0.214	0.135	0.104	0.041
Hypertension	0.162	0.093	0.231	0.162	0.201	0.134	0.097	0.041
Obesity	0.141	0.099	0.212	0.168	0.177	0.141	0.080	0.045
Smoking	0.110	0.105	0.176	0.176	0.146	0.147	0.056	0.048
Asthma	0.109	0.105	0.166	0.177	0.139	0.147	0.059	0.049

Liao LD, *et al. BMJ Open* 2023; 13:e072436. doi: 10.1136/bmjopen-2023-072436

Fig. S1. Flowchart for analytic sample development

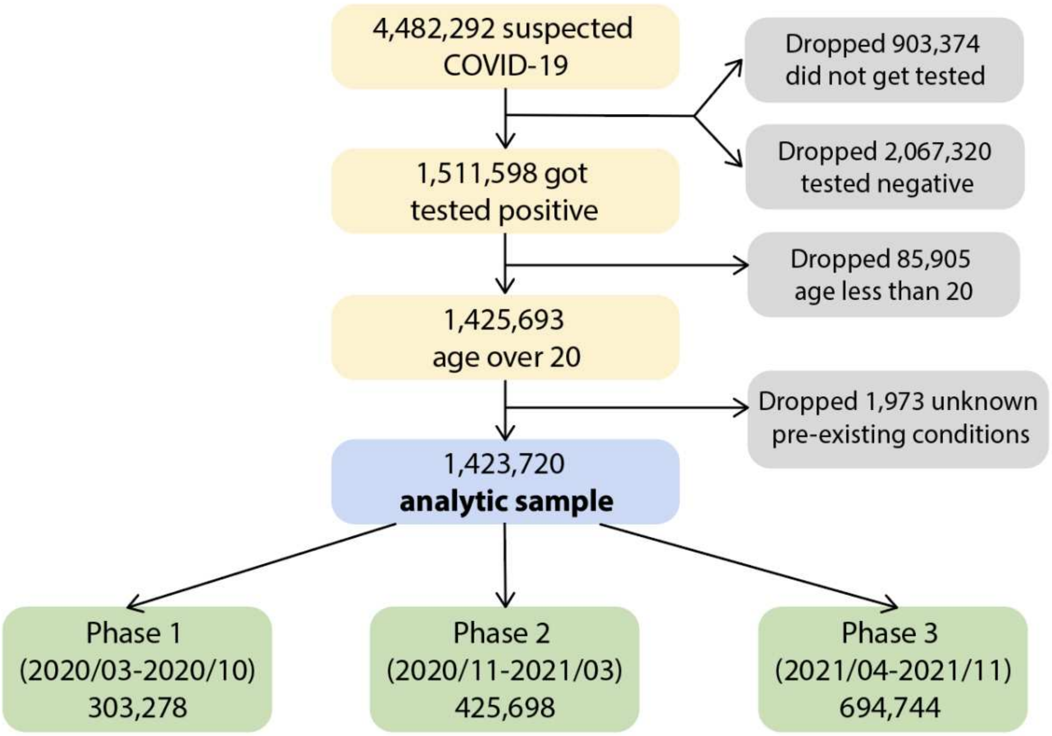

Liao LD, *et al. BMJ Open* 2023; 13:e072436. doi: 10.1136/bmjopen-2023-072436

Fig. S2. Age distribution for laboratory-confirmed COVID-19 patients

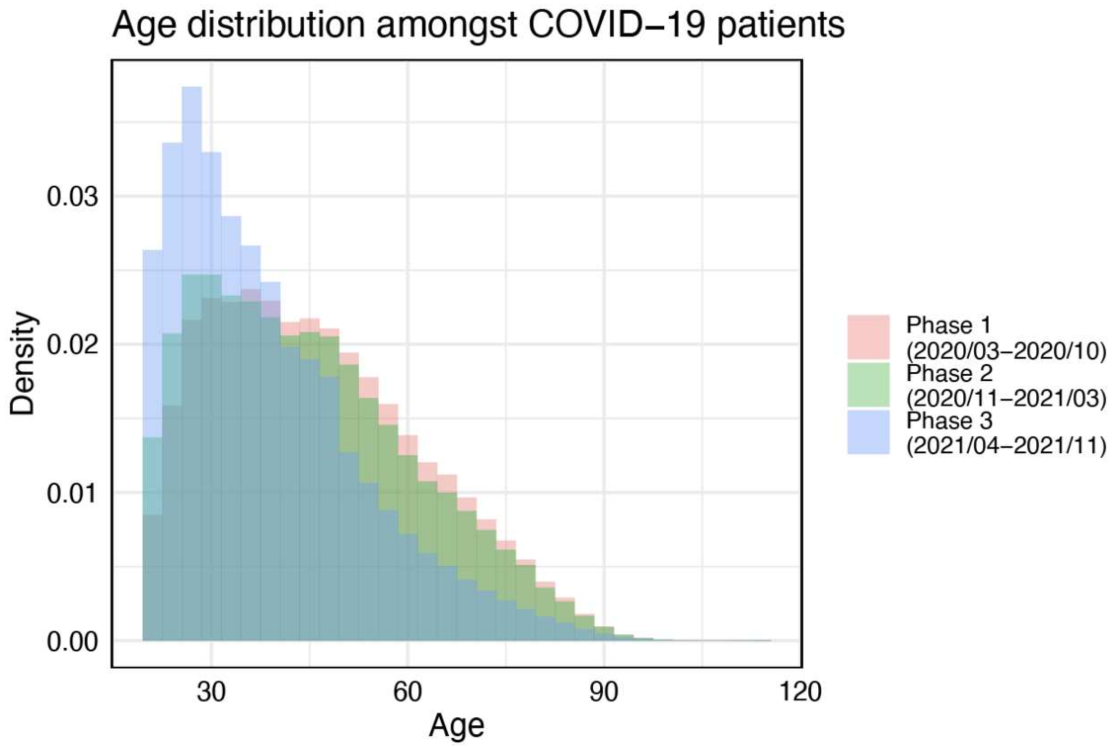

Liao LD, *et al. BMJ Open* 2023; 13:e072436. doi: 10.1136/bmjopen-2023-072436

Fig. S3. Prevalence of pre-existing conditions prevalence over time

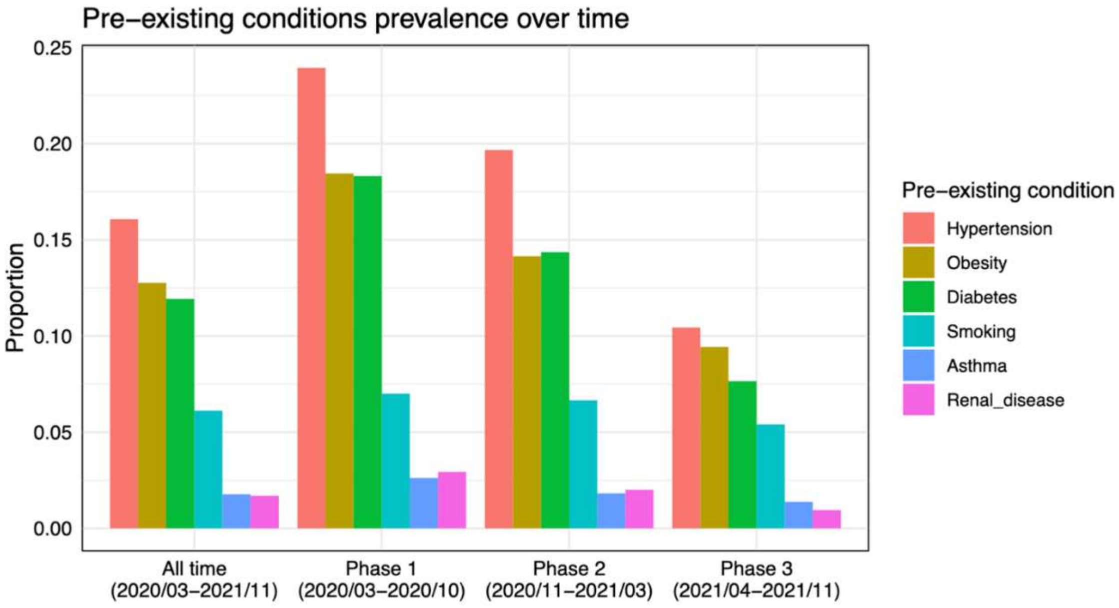

Liao LD, *et al. BMJ Open* 2023; 13:e072436. doi: 10.1136/bmjopen-2023-072436

Fig. S4. Prediction variable importance predicted using the super learner fit

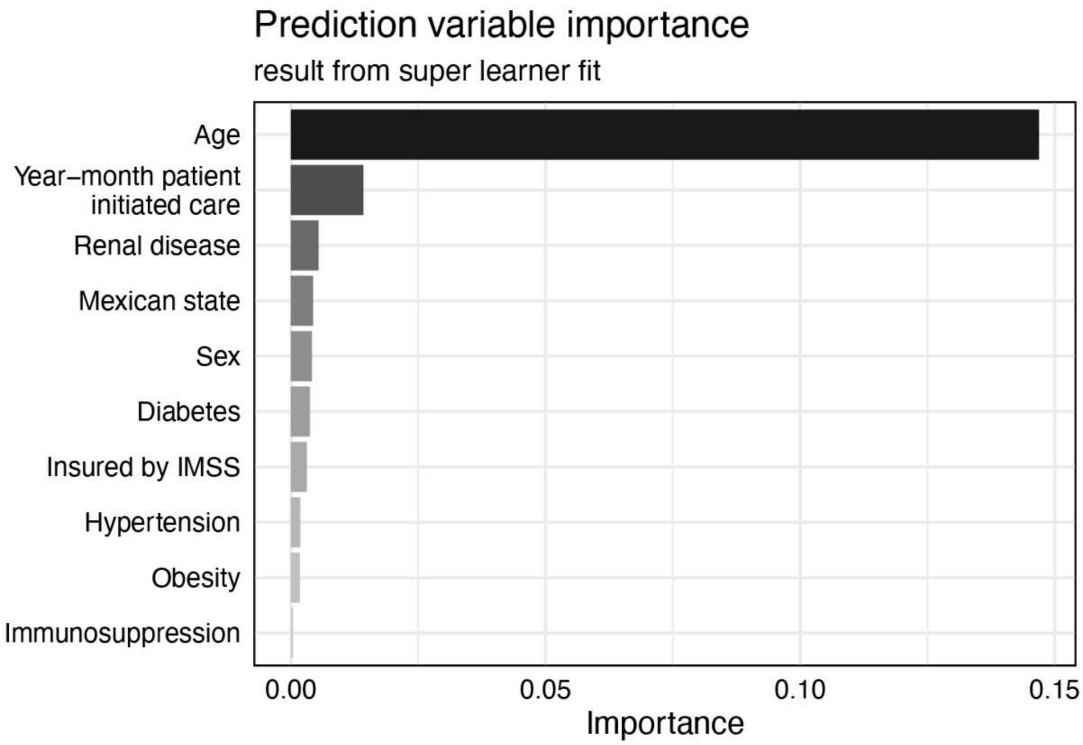

IMSS: Mexican Institute of Social Security

Liao LD, *et al. BMJ Open* 2023; 13:e072436. doi: 10.1136/bmjopen-2023-072436

Fig. S5. Relative risk for each pre-existing condition associated with mortality

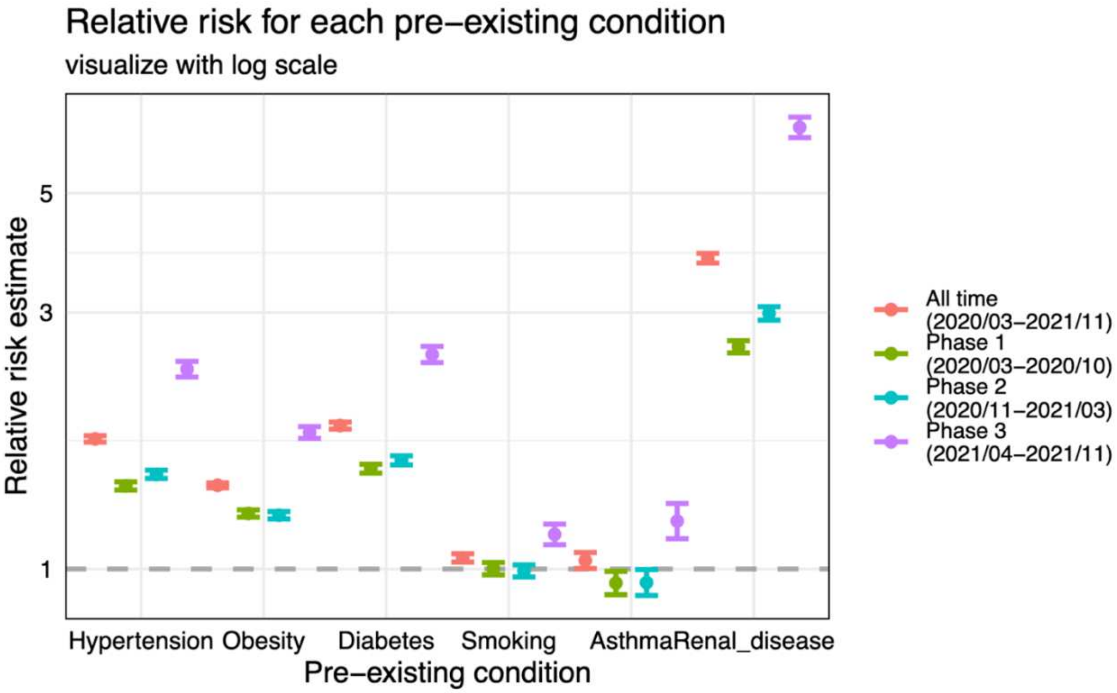

Error bars reflect a 95% confidence interval.

Liao LD, *et al. BMJ Open* 2023; 13:e072436. doi: 10.1136/bmjopen-2023-072436
