# Supplementary material for: Who is most at risk of dying if infected with SARS-CoV-2? A mortality risk factor analysis using machine learning of patients with COVID-19 over time: a large population-based cohort study in Mexico
Source: BMJ Open. 2023 Sep 22;13(9):e072436. doi: 10.1136/bmjopen-2023-072436 (PMC10533798; doi:10.1136/bmjopen-2023-072436)
Supplement: Author's manuscript [file bmjopen-2023-072436.draft_revisions.pdf]

BMJ Open

BMJ Open is committed to open peer review. As part of this commitment we make the peer review history of every article we publish publicly available.

When an article is published we post the peer reviewers' comments and the authors' responses online. We also post the versions of the paper that were used during peer review. These are the versions that the peer review comments apply to.

The versions of the paper that follow are the versions that were submitted during the peer review process. They are not the versions of record or the final published versions. They should not be cited or distributed as the published version of this manuscript.

BMJ Open is an open access journal and the full, final, typeset and author-corrected version of record of the manuscript is available on our site with no access controls, subscription charges or pay-per-view fees (http://bmjopen.bmj.com).

If you have any questions on BMJ Open's open peer review process please email info.bmjopen@bmj.com

BMJ Open

Who is most at risk of dying if infected with SARS-CoV-2? A mortality risk factor analysis using machine learning of COVID-19 patients over time: a large population-based cohort study in Mexico.

Journal:	*BMJ Open*
Manuscript ID	bmjopen-2023-072436
Article Type:	Original research
Date Submitted by the Author:	02-Feb-2023

Complete List of Authors:	Liao, Lauren; University of California Berkeley, Division of Biostatistics<br>Hubbard, Alan; University of California Berkeley, Division of Biostatistics<br>Gutierrez, Juan Pablo; Universidad Nacional Autónoma de México, Center for Policy, Population & Health Research, School of Medicine<br>Juárez, Arturo; Universidad Nacional Autónoma de México<br>Kikkawa, Kendall; Micron Technology<br>Gupta, Ronit; University of California Berkeley<br>Yarmolich, Yana; University of California Berkeley<br>de Jesús Ascencio-Montiel, Iván; Instituto Mexicano del Seguro Social<br>Bertozzi, Stefano; University of California Berkeley School of Public Health; University of Washington School of Public Health

Keywords:	COVID-19, STATISTICS & RESEARCH METHODS, Risk Factors, Hypertension < CARDIOLOGY, General diabetes < DIABETES & ENDOCRINOLOGY, Epidemiology < INFECTIOUS DISEASES

SCHOLARONE™
Manuscripts

I, the Submitting Author has the right to grant and does grant on behalf of all authors of the Work (as defined in the below author licence), an exclusive licence and/or a non-exclusive licence for contributions from authors who are: i) UK Crown employees; ii) where BMJ has agreed a CC-BY licence shall apply, and/or iii) in accordance with the terms applicable for US Federal Government officers or employees acting as part of their official duties; on a worldwide, perpetual, irrevocable, royalty-free basis to BMJ Publishing Group Ltd ("BMJ") its licensees and where the relevant Journal is co-owned by BMJ to the co-owners of the Journal, to publish the Work in this journal and any other BMJ products and to exploit all rights, as set out in our licence.

The Submitting Author accepts and understands that any supply made under these terms is made by BMJ to the Submitting Author unless you are acting as an employee on behalf of your employer or a postgraduate student of an affiliated institution which is paying any applicable article publishing charge ("APC") for Open Access articles. Where the Submitting Author wishes to make the Work available on an Open Access basis (and intends to pay the relevant APC), the terms of reuse of such Open Access shall be governed by a Creative Commons licence – details of these licences and which Creative Commons licence will apply to this Work are set out in our licence referred to above.

Other than as permitted in any relevant BMJ Author's Self Archiving Policies, I confirm this Work has not been accepted for publication elsewhere, is not being considered for publication elsewhere and does not duplicate material already published. I confirm all authors consent to publication of this Work and authorise the granting of this licence.

For peer review only

Who is most at risk of dying if infected with SARS-CoV-2? A mortality risk factor analysis using machine learning of COVID-19 patients over time: a large population-based cohort study in Mexico.

Lauren D. Liao,[1*] Alan E. Hubbard,[1] Juan Pablo Gutiérrez,[2] Arturo Juárez-Flores,[2] Kendall Kikkawa,[3] Ronit Gupta,[1] Yana Yarmolich,[1] Iván de Jesús Ascencio-Montiel,[4] Stefano M. Bertozzi[1,5,6]

Affiliations:

[1] University of California, Berkeley, School of Public Health, Berkeley, CA, USA

[2] Center for Policy, Population & Health Research, School of Medicine, Universidad Nacional Autónoma de México, Mexico City, Mexico

[3] Micron Technology, Boise, ID, USA

[4] Instituto Mexicano del Seguro Social, CDMX, México

[5] University of Washington School of Public Health, Seattle, WA, USA

[6] Instituto Nacional de Salud Pública, Cuernavaca, MOR, México

***Correspondence to:** ldliao@berkeley.edu (LDL)

Word count: 2884

For peer review only - http://bmjopen.bmj.com/site/about/guidelines.xhtml

Abstract

Objective: COVID-19 would kill fewer people if health programs can predict who is at higher risk of mortality because resources can be targeted to protect those people from infection. We predict mortality in a very large population in Mexico with machine learning using demographic variables and pre-existing conditions.

Design Cohort study.

Setting March 2020 to November 2021 in Mexico, nationally represented.

Participants 1.4 million laboratory-confirmed COVID-19 patients in Mexico at or over 20 years of age.

Primary and secondary outcome measures Analysis is performed on data from March 2020 to November 2021 and over three phases: (1) from March to October in 2020, (2) from November 2020 to March 2021, and (3) from April to November 2021. We predict mortality using an ensemble machine learning method, super learner, and independently estimate the adjusted mortality relative risk of each pre-existing condition using targeted maximum likelihood estimation.

Results Super learner fit has a high predictive performance (C-statistic: 0.907), where age is the most predictive factor for mortality. After adjusting for demographic factors, renal disease, hypertension, diabetes, and obesity are the most impactful pre-existing conditions. Phase analysis shows that the adjusted mortality risk decreased over time while relative risk increased for each pre-existing condition.

Conclusions While age is the most important predictor of mortality, younger individuals with hypertension, diabetes and obesity are at comparable mortality risk as individuals who are 20

For peer review only - http://bmjopen.bmj.com/site/about/guidelines.xhtml

years older without any of the three conditions. Our model can be continuously updated to identify individuals who should most be protected against infection as the pandemic evolves.

Keywords: COVID-19; Epidemiology; Diabetes; Hypertension; Statistical methods; Risk Factors

For peer review only

Strengths and limitations of this study

- Compared to previous studies showing age and the presence of pre-existing conditions as important predictors of mortality, our study leveraged more powerful statistical approaches by examining mortality risk in a very large population (> 60 M).
- Analyses based on a combination of machine learning and causal inference methods for prediction and estimation of risk factor impacts that is robust to model misspecification.
- The phase analyses presented in this study demonstrates marked changes over time in the degree to which different risk factors predict mortality.
- Preexisting conditions are self-reported, and thus subject to potential misspecification.
- Those who are included in the data may not be representative of the socioeconomic status of the entire population; thus, extrapolation of the result should be done with caution.

For peer review only

For peer review only - http://bmjopen.bmj.com/site/about/guidelines.xhtml

Introduction

The probability that someone infected with SARS-CoV-2 dies has varied enormously over time, among countries, and among population groups within countries. Interest in understanding who is at a higher risk of death has grown as this heterogeneity became more apparent. Identifying people at higher risk of severe disease and death will help health systems better respond and focus prevention resources on protecting them. We examine Mexico, a country with a very high reported case-fatality rate (4.7%) among those who have laboratory-confirmed coronavirus disease 2019 (COVID-19) as of September 23, 2022 [1].

Previous analyses in Mexico have found diabetes, obesity, hypertension, immunosuppression, and renal disease to be significant risk factors along with age and sex. Multiple authors have identified obesity and diabetes as important risk factors for mortality [2–5]. Escobedo de-la Peña et al. also found a strong association with hypertension, which is consistent with results from Giannouchos et al. [5,6]. Late-stage chronic kidney disease, although less prevalent, has also consistently been identified as a COVID-19 mortality risk factor . Older/ male patients tend to have higher mortality risks than younger/ female patients [3,5,6]. In a previous analysis, we found interactions between those comorbidities, suggesting a synergic effect when having more than one of diabetes, hypertension, and obesity (larger odds ratio when reporting the 3 conditions vs. one or two) [7]. We also found that the odds ratio increased by age group with those over age 80 having 30-fold the risk of those 20 to 29 [7]. One important consideration is that the prevalence of diabetes and hypertension is positively associated with age, so it has not been clear how this interaction is related to mortality risk. A more adaptive analysis performed by Martínez-Martínez et al. developed a prediction model for severity of COVID-19, defined by hospitalization and/or mortality. They examined the relationship of 14

For peer review only - http://bmjopen.bmj.com/site/about/guidelines.xhtml

variables with hospitalization and mortality using interaction terms and splines to account for non-linear relationships [8].

The pattern of age, sex, and comorbidities being associated with higher mortality risk is not specific to Mexico, and the global literature on such associations is extensive. Researchers have identified old age, diabetes, obesity, chronic renal failure, and congestive heart failure to be strongly associated with severe infection amongst both sexes in the Spanish population [9]. Researchers in Brazil showed that older age, male, kidney disease, obesity and/ or diabetes are strong predictors of mortality amongst other comorbidities such as chronic liver disease, immunosuppression, and cardiovascular disease [10,11]. Another study used United Kingdom Biobank data and showed that pre-existing dementia, diabetes, chronic obstructive pulmonary disease (COPD), pneumonia, and depression were positively associated with risk of hospitalization and death [12]. An analysis from France found age, diabetes, hypertension, obesity, cancer, and kidney and lung transplants to be associated with risk of COVID-19-related hospitalization and mortality, among others [13]. A Canadian study reported dementia, chronic kidney disease, cardiovascular disease, diabetes, COPD, severe mental illness, organ transplant, hypertension, and cancer to be significant predictors of mortality [14]. Our goal in this study is not only to predict mortality using demographic factors and comorbidities, but to show how those predictions change over time in this rapidly evolving pandemic.

Although mortality risk estimation and risk factor identification have been examined in prior studies, we are concerned about the statistical validity and interpretation of the standard methods. A commonly used prediction tool, logistic regression, assumes a linear relationship of predictors against the log odds of mortality risk, but this logit-linear assumption will lead inevitably to biased estimates of risk (either under- or over-predict the risk) for subsets of the

For peer review only - http://bmjopen.bmj.com/site/about/guidelines.xhtml

population. We instead used flexible, data-adaptive methods that can capture non-linearities in the dose-response, such as potential nonlinear interactions between the predictors (e.g., the potential interaction of age and diabetes on predicting death) [15,16]. The better the model fits the study population; the more likely estimates are closer to the true joint relationship of mortality and risk factors.

We included pre-existing conditions, demographic variables, the Mexican state where the patient was treated, and the month that the patient initiated care to fit our prediction algorithm. We conducted the analysis using an ensemble machine learning algorithm, super learner, to form optimal combination of predictions from multiple machine learning methods [15,16]. We also estimated the comparative importance of variables for mortality risk prediction (holding all other variables constant) by nonparametrically estimating quantities inspired by causal parameters (parameters that compare so-called counterfactual distributions, in our case, causal relative risks). The statistical goal is to estimate and provide robust inference for impact estimates of the predictors without the arbitrary modeling assumptions that characterize the great majority of prior work [17].

Methods

Study population and design

The study population is drawn from the Mexican Social Security Institute (IMSS), a vertically integrated insurance and health system that provides coverage for over 60 million private sector employees and their families, including their parents, children and spouse. IMSS also provided care as part of the COVID-19 response for some non-beneficiaries, who are also

For peer review only - http://bmjopen.bmj.com/site/about/guidelines.xhtml

included in the dataset. No patient or the public were involved in the design, or conduct, or reporting, or dissemination plans of our research.

The data were recorded from March 1st, 2020, to November 3rd, 2021 in a platform called SINOLAVE. They reflect the entire population of 4,482,292 patients who were registered as receiving care for suspected COVID-19 at an IMSS facility. The dataset and the data entry process have been described previously [18]. The demographic variables include age, sex, insured by IMSS, and indigenous status. The data contains pre-existing conditions reported by the patient or the family at presentation: asthma, cardiovascular disease, chronic liver disease, chronic obstructive pulmonary disease, diabetes, hemolytic anemia, human immunodeficiency virus, hypertension, immunosuppression, neurological disease, obesity, cancer, renal disease and tuberculosis, as well as whether the patient currently smokes. Patients were asked at presentation about their pre-existing health conditions; these were not ascertained with reference to the patient's medical record, even for those patients insured by the IMSS. The data also includes the Mexican state in which the patient received care, COVID-19 test results (from both polymerase chain reaction (PCR) tests and antigen tests), the month that the patient initiated care, and mortality. In addition, we extracted a different dataset from the National Council of Science and Technology to determine the dominant circulating variant in each month [19]. A short summary can be found in **Table 1 (Supplemental Table S1)**. We define COVID-19 positive as a positive PCR or antigen test.

For peer review only

Table 1. Summary table of baseline variables and pre-existing conditions

	**All time** (2020/03-2021/11)	**Phase 1** (2020/03-2020/10)	**Phase 2** (2020/11-2021/03)	**Phase 3** (2021/04-2021/11)
Sample size	1,423,720	303,278	425,698	694,744
**Demographic variables**				
Age in years (mean (SD))	42.15 (15.70)	46.41 (16.04)	44.89 (16.27)	38.61 (14.34)
Sex = male (%)	729,782 (51.3)	158,248 (52.2)	218,165 (51.2)	353,369 (50.9)
Insured by IMSS = yes (%)	1,358,440 (95.4)	288,588 (95.2)	402,754 (94.6)	667,098 (96.0)
Indigenous = yes (%)	7,381 (0.5)	2,200 (0.7)	1,628 (0.4)	3,553 (0.5)
**Pre-existing conditions**				
Hypertension = yes (%)	228,901 (16.1)	72,615 (23.9)	83,735 (19.7)	72,551 (10.4)
Diabetes = yes (%)	169,869 (11.9)	55,551 (18.3)	61,120 (14.4)	53,198 (7.7)
Obesity = yes (%)	181,736 (12.8)	55,965 (18.5)	60,217 (14.1)	65,554 (9.4)
Smoking = yes (%)	87,161 (6.1)	21,253 (7.0)	28,346 (6.7)	37,562 (5.4)
Asthma = yes (%)	25,297 (1.8)	7,951 (2.6)	7,765 (1.8)	9,581 (1.4)
Renal Disease Diagnosis = yes (%)	24,099 (1.7)	8,912 (2.9)	8,555 (2.0)	6,632 (1.0)
**Outcome**				
Death = yes (%)	149,805 (10.5)	53,530 (17.7)	62,517 (14.7)	33,758 (4.9)

IMSS: Mexican Institute of Social Security; SD: standard deviation

From the full data set, we generated an analytic sample (n = 1,423,720) **(Supplemental Figure S1)**. We exclude those under the age of 20 years, those without any positive COVID-19 test result from either the PCR or antigen tests, and those with unknown pre-existing conditions. We also create a phase variable that corresponds to changes in the epidemic curve into three: phase 1 is from March 1st, 2020, to October 31st, 2020, phase 2 is from November 1st, 2020, to

For peer review only - http://bmjopen.bmj.com/site/about/guidelines.xhtml

March 31st, 2021, and phase 3 is from April 1st, 2021, to November 3rd, 2021 as previously described [18].

Statistical analysis

Mortality risk prediction using super learner (SL)

We predict mortality risks with SL [15,16], using predictors: pre-existing conditions, demographic variables, the Mexican state where the patient was treated, and the month that the patient initiated care. SL combines a set of user-supplied machine learning algorithms, which includes both simple, parametric fits and flexible algorithms, to create an optimally-weighted combination. This optimal fit is found by creating a combination of algorithms that minimize the cross-validated risk (in our case, the negative log-likelihood). SL has the property that asymptotically it will perform at least as well as the best fitting algorithm in the library [15,16]. Thus, it is important to include a diverse and large set of learners as candidates to ensure the model can fit complex patterns if warranted, but also, simpler, parametric models if simpler fits are sufficient. The following learners were included in the SL library: Bayesian additive regression trees [21], Bayesian generalized linear model [22], elastic net regression [23], empirical mean, generalized additive model [24], least absolute shrinkage and selection operator regression [25], logistic regression, multivariate adaptive regression splines [26], random forest [27], ridge regression [28], and extreme gradient boosting algorithms [29]. We estimate the prediction performance, via the AUC, and derive a 95% confidence interval for the estimated AUC [30]. We compare the SL fit using all predictors listed above to a logistic regression with only age entered as a linear term. We compute the AUC for the resulting SL/logistic regression fits with 3-fold cross validation on the 80%, both on the same data used to estimate SL/logistic

For peer review only - http://bmjopen.bmj.com/site/about/guidelines.xhtml

regression models (training AUC), as well as a more realistic assessment by using the test set – the left-out 20% of the available data (testing AUC).

To interpret the final prediction model generated by the SL fit, we use the permutation-based variable importance measure to identify variables that influence the SL model's prediction [27]. This is performed by permuting the predictor variables one at a time (keeping the other variables fixed) and measuring the magnitude of the decline on the predictive performance (as measured by the change in the average negative log-likelihood). This provides a list of variables ranked by the relative importance to prediction fit but does not provide information on the variable impact on mortality, which led us to another measure of relative risk (RR) using targeted maximum likelihood estimation (TMLE).

Pre-existing condition relative risk estimate through targeted maximum likelihood estimation

For pre-existing conditions, we estimated a different variable importance measure that is not focused on prediction accuracy but on estimating potential impacts of pre-existing conditions on mortality risk. The impact is estimated by the RR of adjusted means (adjusted for baseline confounders) for the population if everyone had the specific pre-existing condition of interest (the numerator) versus the same population where no one has the specific pre-existing condition (the denominator). To estimate RRs, we used cross-validated targeted minimum-loss-based estimation (cross-validated TMLE). TMLE is a semiparametric, substitution estimator that has shown to be asymptotically efficient (unlike the inverse probability of treatment-weighting estimators [31]). It also has some robustness advantages over other semiparametric efficient approaches, such as augmented inverse probability weighting. TMLE estimates parameters that,

For peer review only - http://bmjopen.bmj.com/site/about/guidelines.xhtml

under certain assumptions, can be interpreted as potential causal impacts of these factors on mortality, in our case, in the form of a causal relative risk. Our ensemble machine learning is optimized for prediction, but it does not directly provide measures of individual variable importance. We conducted follow-up procedure (TMLE) to generate interpretable estimates of variable impact with robust standard errors [32, 33].

Results

Descriptive results show the age distribution of laboratory-confirmed patients across the three different epidemic phases **(Supplemental Figure S2**). Phases 1 and 2 have similar distributions, and there are more young people (under 30) in phase 3. The six most prevalent pre-existing conditions are hypertension, obesity, diabetes, smoking, asthma, and renal disease **(Supplemental Figure S3**). The prevalence of all pre-existing conditions decreased over the three phases, and prevalence of hypertension, obesity, and diabetes were drastically reduced in phase 3.

Super learner (SL) prediction

SL fit has high prediction accuracy on the testing set (AUC: 0.907 (95% CI: (0.905-0.908)). SL leverages XGBoost models (**Supplemental Table S2)** and significantly outperforms the simple logistic regression model (testing AUC: 0.874 (95% CI: (0.872-0.876)) (**Table 2**). The logistic regression model overpredicts mortality risks for those roughly above age 75 compared to the SL prediction (**Fig. 1**). Permuted variable importance shows, while holding other variables constant, age is consistently the most important for SL prediction in average mortality risk (**Supplemental Figure S4 and Table S3)**. Having multiple comorbidities can dramatically increase risk for those individuals (**Fig. 2**).

For peer review only - http://bmjopen.bmj.com/site/about/guidelines.xhtml

Table 2. Prediction results

	All time (2020/03-2021/11) AUC (95% CI)	Phase 1 (2020/03-2020/10) AUC (95%CI)	Phase 2 (2020/11-2021/03) AUC (95%CI)	Phase 3 (2021/04-2021/11) AUC (95%CI)
Super learner fit	Training: 0.916 (0.915-0.917)<br><br>Testing: 0.907 (0.905-0.908)	Training: 0.887 (0.885-0.888)<br><br>Testing: 0.873 (0.870-0.876)	Training: 0.904 (0.903-0.906)<br><br>Testing: 0.895 (0.892-0.897)	Training: 0.914 (0.913-0.916)<br><br>Testing: 0.906 (0.902-0.909)
Age only logistic regression fit	Training: 0.874 (0.873-0.875)<br><br>Testing: 0.874 (0.872-0.876)	Training: 0.845 (0.843-0.846)<br><br>Testing: 0.846 (0.842-0.850)	Training: 0.868 (0.866-0.870)<br><br>Testing: 0.871 (0.868-0.874)	Training: 0.867 (0.865-0.869)<br><br>Testing: 0.871 (0.866-0.875)

AUC: area under the receiver operating characteristic curve; CI: confidence interval

224

225 **Relative risks of pre-existing conditions**

226 To assess the impact of each pre-existing condition, we estimate their respective relative
227 risks (RRs) of mortality, adjusting for demographic variables. We report the estimated RRs in
228 **Table 3**, ordered by impact (most to least) (**Supplemental Figure S5**). The RRs compare the
229 expected risk if all patients have the pre-existing condition (with) versus if all patients do not
230 have the condition (without). The highest impact pre-existing condition is renal disease (RR:
231 3.783, 95% CI: (3.705, 3.862)); diabetes, obesity, and hypertension also have high impact
232 individually (RR: 1.432-1.847). Minimal differences between the risk estimates are shown for
233 smoking and asthma (RR: 1.049 and 1.037, respectively).

234

For peer review only - http://bmjopen.bmj.com/site/about/guidelines.xhtml

Table 3. Targeted maximum likelihood estimation relative risk results for each pre-existing condition

	All time (2020/03-2021/11) Relative Risk (95% CI)	Phase 1 (2020/03-2020/10) Relative Risk (95%CI)	Phase 2 (2020/11-2021/03) Relative Risk (95%CI)	Phase 3 (2021/04-2021/11) Relative Risk (95%CI)
Renal disease	3.783 (3.705, 3.862)	2.588 (2.521, 2.657)	2.994 (2.910, 3.080)	6.638 (6.361, 6.927)
Diabetes	1.847 (1.820, 1.875)	1.536 (1.508, 1.566)	1.594 (1.564, 1.625)	2.508 (2.423, 2.596)
Hypertension	1.745 (1.721, 1.770)	1.427 (1.402, 1.452)	1.500 (1.474, 1.527)	2.356 (2.279, 2.436)
Obesity	1.432 (1.417, 1.447)	1.269 (1.249, 1.288)	1.259 (1.239, 1.279)	1.794 (1.750, 1.840)
Smoking	1.049 (1.030, 1.068)	1.001 (0.975, 1.028)	0.992 (0.966, 1.018)	1.158 (1.107, 1.210)
Asthma	1.037 (1.002, 1.073)	0.941 (0.895, 0.989)	0.942 (0.892, 0.995)	1.223 (1.134, 1.319)

CI: confidence interval

The phase analyses indicate pre-existing conditions are especially important in phase 3. Phase 1 and 2 are very similar in terms of both risk prediction and adjusted mortality risk estimates. However, in phase 3, age is less important in prediction (**Supplemental Table S3)** and RRs drastically increase for every comorbidity. The adjusted risks show the decrease for each pre-existing condition in phase 3 (**Supplemental Table S4)**.

For peer review only - http://bmjopen.bmj.com/site/about/guidelines.xhtml

Discussion

Our analysis of (>1.4 million) laboratory-confirmed COVID-19 patients demonstrates that age is by far the most important predictor of average mortality. For those patients with renal disease, diabetes, hypertension, or obesity, having the comorbidity further increases their risk of mortality. A patient with diabetes, hypertension, and obesity is roughly comparable to a patient 20 years older with none of the conditions, based on the predicted mortality (**Fig. 2**). Thus, having a comorbidity increases risk of mortality and should be considered at any age. The reason that comorbidities add little to the predictive power at younger ages is that hypertension and diabetes are age-related and the reported onset is often for those over 30, so the pre-existing conditions are far less prevalent.

Our prediction results using machine learning methods predict better than previous studies, and we demonstrated the feasibility and robustness of using machine learning methods targeted for prediction and variable impact. SL model prediction has an AUC of 0.907, which is higher than any previous Mexican study (AUCs from 0.634 to 0.824) [8,34]. Although age has been well reported by previous studies as important [5,34,35], our analysis is more robust because we do not assume a pre-specified functional relationship between the explanatory variables and the predicted variable, and thereby avoid any arbitrary groupings into age categories. Moreover, since those above age 60 have a higher prevalence of comorbidities, relying on simple logistic regression models can greatly overpredict the average mortality risk for the elder patients. Our study applies TMLE to estimate the adjusted mortality risk ratios for each comorbidity to provide more robust impact estimates that respect time ordering and account for background variables.

For peer review only - http://bmjopen.bmj.com/site/about/guidelines.xhtml

We find consistent results of comorbidities compared to previous studies, and present phase analyses highlighting the changes in relative risks over time. Previous results from logistic regressions indicated odds ratios of 1.458-2.48 for renal disease, 1.237-1.74 for diabetes, 1.173-1.47 for obesity, 1.194-1.315 for hypertension, 0.852-1.02 for smoking, and 0.74-1.420 for asthma [34–36]. Although our analysis is generally consistent with previous findings, our RR estimations have less uncertainty. Renal disease has the greatest impact on mortality, followed by diabetes, hypertension, and obesity; smoking and asthma have negligible impact on mortality risk.

This phase-specific analysis produced a seemingly paradoxical finding. The impact of comorbidities on predicted mortality decreased with time (primarily between the second and third wave), but the RR on mortality dramatically increased for the same conditions **(Supplemental Table S4 and Figure S5)**. The apparent explanation is that mortality risk for people without the comorbidities fell faster than for people with them, increasing the relative risk. The decrease in mortality risk is multifactorial and includes a decrease in susceptibility over time (due to prior infection and vaccination), improved treatment, enhanced healthcare response and opportunity to be admitted to a hospital or ICU, and less virulent viral subtypes. This implies that as herd immunity increases, medical resources should focus even more on protecting vulnerable people at older age and those with comorbidities since they are even more likely to experience severe outcomes compared to those who are younger and/or healthier.

Readers should be cautious about extrapolating our findings to other populations. Although our sample is large and includes patients from all parts of Mexico, most of the patients were IMSS beneficiaries. In order to access IMSS health services, patients require: a) be a formal-sector worker or retired, b) be a direct dependent of such an employee, c) be a bachelor or

For peer review only

For peer review only - http://bmjopen.bmj.com/site/about/guidelines.xhtml

postgraduate student in a public institution, d) voluntarily enroll by paying a fee. Thus, the IMSS population skews toward the upper half of the income distribution. Populations without similar access to health services may have different results. It is also important to consider the potential impact of data quality. Pre-existing conditions were self-reported and likely also inconsistently recorded, perhaps in systematic ways that could have biased the results. For example, if people with severe diabetes were more likely to report diabetes as a pre-existing condition, we may overestimate the impact of diabetes on mortality.

It is also important to consider what predictive variables are included in this model. We sought to predict risk for an individual in the population using their characteristics prior to infection. In other words, what is this person's risk of death from COVID-19 if they were to be infected? The answer to this question best informs the question of who should be prioritized for protection against infection or for early therapeutic interventions following infection. It does not attempt to predict the likely mortality of a patient who presents to the health services with COVID-19 because information about that patient's severity of their COVID-19-related symptoms will represent important additional predictors of their mortality risk.

For peer review only - http://bmjopen.bmj.com/site/about/guidelines.xhtml

Abbreviations

AUC, area under the receiver operating characteristic curve; CI, confidence interval; COPD, chronic obstructive pulmonary disease; COVID-19, coronavirus disease of 2019; IMSS, Mexican Social Security Institute; PCR, polymerase chain reaction; RR, relative risk; SL, super learner; TMLE, targeted maximum likelihood estimation; XGBoost, extreme gradient boosting

Declarations:

Acknowledgements

We thank the staff of C3.ai DTI for their technical support and our colleagues at University of California, Berkeley, the Mexican National Autonomous University, and the Mexican Social Security Institute (IMSS) for all of the administrative and technical support that has allowed this collaboration to flourish.

Authors' contributions

LDL and AEH contributed to the study design and methodology. AJ, YY, and IA contributed to data acquisition. LDL, YY, and KK contributed to data cleaning. LDL led the data analysis and visualization. LDL, AEH, JPG, and SMB interpreted the results. LDL drafted the manuscript with support from RG on literature search. AEH and SMB significantly contributed to the revision of the manuscript. All authors participated in review and edited the manuscript; all authors have read and approved the final manuscript. All authors had full access to all the data in the study and accepted responsibility to submit for publication. All authors take responsibility for the integrity of the data and the accuracy of the data analysis.

For peer review only

For peer review only - http://bmjopen.bmj.com/site/about/guidelines.xhtml

Funding

This research effort was funded by the C3.ai Digital Transformation Institute. The C3.ai DTI was established by C3.ai, Microsoft, the University of California, Berkeley (UC Berkeley), the University of Illinois at Urbana-Champaign (UIUC), Carnegie Mellon University, University of Chicago, MIT, and Princeton University. It is being funded in cash and in kind by C3.ai, Microsoft Azure, and the Lawrence Berkeley National Laboratory. The funders had no role in access to data, design of the research, or analyses conducted. They have not seen or contributed to the manuscript in any way.

In addition, LDL received funding from the National Science Foundation (DGE 2146752). AEH received funding from a global development grant (OPP1165144) from the Bill & Melinda Gates Foundation to the University of California, Berkeley, CA, USA.

For peer review only

Competing interests

All authors declare no competing interests.

Patient consent for publication

Not required.

Patient and Public Involvement

Patients or the public were not involved in the design, or conduct, or reporting, or dissemination plans of our research

Ethics approval

This data-only study was approved on November 4th, 2020, by the Scientific Research National Committee (Social Security Mexican Institute) with R-2020-785-165. The University of

For peer review only - http://bmjopen.bmj.com/site/about/guidelines.xhtml

California, Berkeley Institutional Review Board (IRB) determined that the project was exempt from IRB approval.

Availability of data and materials

The study was conducted using confidential patient records subject to strict access controls and we are therefore unable to share the data that were used for this study.

Consent for publication

Not applicable.

For peer review only

For peer review only - http://bmjopen.bmj.com/site/about/guidelines.xhtml

References

1. Hopkins. University of Medicine. Coronavirus Resource Center. *Data Stream* 2020.

2. Singer M. Deadly Companions: COVID-19 and Diabetes in Mexico. *Med Anthropol* 2020;**39**:660–5.

3. Bello-Chavolla OY, Bahena-López JP, Antonio-Villa NE, *et al.* Predicting Mortality Due to SARS-CoV-2: A Mechanistic Score Relating Obesity and Diabetes to COVID-19 Outcomes in Mexico. *J Clin Endocrinol Metab* 2020;**105**. doi:10.1210/clinem/dgaa346

4. Noyola DE, Hermosillo-Arredondo N, Ramírez-Juárez C, *et al.* Association between obesity and diabetes prevalence and COVID-19 mortality in Mexico: an ecological study. *J Infect Dev Ctries* 2021;**15**:1396–403.

5. Giannouchos TV, Sussman RA, Mier Odriozola JM, *et al.* Characteristics and risk factors for COVID-19 diagnosis and adverse outcomes in Mexico: an analysis of 89,756 laboratory–confirmed COVID-19 cases. bioRxiv. 2020. doi:10.1101/2020.06.04.20122481

6. Peña JE la, Rascón-Pacheco RA, Ascencio-Montiel I de J, *et al.* Hypertension, Diabetes and Obesity, Major Risk Factors for Death in Patients with COVID-19 in Mexico. *Arch Med Res* 2021;**52**:443–9.

7. Gutierrez JP, Bertozzi SM. Non-communicable diseases and inequalities increase risk of death among COVID-19 patients in Mexico. *PLoS One* 2020;**15**:e0240394.

8. Martínez-Martínez MU, Alpízar-Rodríguez D, Flores-Ramírez R, *et al.* An Analysis COVID-19 in Mexico: a Prediction of Severity. *J Gen Intern Med* 2022;**37**:624–31.

9. Gimeno-Miguel A, Bliek-Bueno K, Poblador-Plou B, *et al.* Chronic diseases associated with increased likelihood of hospitalization and mortality in 68,913 COVID-19 confirmed cases in Spain: A population-based cohort study. *PLoS One* 2021;**16**:e0259822.

10. Soares R de CM, Mattos LR, Raposo LM. Risk Factors for Hospitalization and Mortality due to COVID-19 in Espírito Santo State, Brazil. *Am J Trop Med Hyg* 2020;**103**:1184–90.

11. Wollenstein-Betech S, Silva AAB, Fleck JL, *et al.* Physiological and socioeconomic characteristics predict COVID-19 mortality and resource utilization in Brazil. *PLoS One* 2020;**15**:e0240346.

12. Atkins JL, Masoli JAH, Delgado J, *et al.* Preexisting Comorbidities Predicting COVID-19 and Mortality in the UK Biobank Community Cohort. *J Gerontol A Biol Sci Med Sci* 2020;**75**:2224–30.

13. Semenzato, Botton, Drouin, *et al.* Chronic diseases, health conditions and risk of COVID-19-related hospitalization and in-hospital mortality during the first wave of the epidemic in France: a …. *The Lancet Regional* https://www.sciencedirect.com/science/article/pii/S2666776221001356

For peer review only - http://bmjopen.bmj.com/site/about/guidelines.xhtml

14. Ge E, Li Y, Wu S, *et al.* Association of pre-existing comorbidities with mortality and disease severity among 167,500 individuals with COVID-19 in Canada: A population-based cohort study. *PLoS One* 2021;**16**:e0258154.

15. van der Laan MJ, Polley EC, Hubbard AE. Super learner. *Stat Appl Genet Mol Biol* 2007;**6**:Article25.

16. Polley EC, van der Laan MJ. Super Learner In Prediction. Published Online First: 2010.https://biostats.bepress.com/ucbbiostat/paper266/?TB_iframe=true&width=370.8&height=658.8 (accessed 26 Jul 2022).

17. Petersen ML, van der Laan MJ. Causal models and learning from data: integrating causal modeling and statistical estimation. *Epidemiology* 2014;**25**:418–26.

18. Juárez-Flores A, Ascencio-Montiel IJ, Gutiérrez JP, *et al.* COVID-19 in the Mexican Social Security Institute (IMSS) population. Prevalent symptoms. bioRxiv. 2022. doi:10.1101/2022.04.12.22273734

19. Vigilancia de variantes del virus SARS-CoV-2. Vigilancia de variantes del virus SARS-CoV-2. https://salud.conacyt.mx/coronavirus/variantes/. (accessed 29 Jul 2022).

20. Hubbard AE, Kennedy CJ, van der Laan MJ. Data-Adaptive Target Parameters. In: van der Laan MJ, Rose S, eds. *Targeted Learning in Data Science: Causal Inference for Complex Longitudinal Studies*. Cham: : Springer International Publishing 2018. 125–42.

21. Chipman HA, George EI, McCulloch RE. BART: Bayesian additive regression trees. *aoas* 2010;**4**:266–98.

22. Dey DK, Ghosh SK, Mallick BK. *Generalized linear models: A Bayesian perspective*. CRC Press 2000.

23. Hans C. Elastic Net Regression Modeling With the Orthant Normal Prior. *J Am Stat Assoc* 2011;**106**:1383–93.

24. Liu. Generalized additive model. *Rep Univ Jyvaskyla Dep Math Stat* Published Online First: 2008.http://people.vcu.edu/~dbandyop/BIOS625/GAM.pdf

25. Tibshirani R. Regression Shrinkage and Selection via the Lasso. *J R Stat Soc Series B Stat Methodol* 1996;**58**:267–88.

26. Friedman JH. Multivariate Adaptive Regression Splines. *aos* 1991;**19**:1–67.

27. Breiman L. Random Forests. *Mach Learn* 2001;**45**:5–32.

28. Hoerl AE, Kennard RW. Ridge Regression: Biased Estimation for Nonorthogonal Problems. *Technometrics* 1970;**12**:55–67.

For peer review only

For peer review only - http://bmjopen.bmj.com/site/about/guidelines.xhtml

29. Chen T, Guestrin C. XGBoost: A Scalable Tree Boosting System. In: *Proceedings of the 22nd ACM SIGKDD International Conference on Knowledge Discovery and Data Mining*. New York, NY, USA: : Association for Computing Machinery 2016. 785–94.

30. LeDell E, Petersen M, van der Laan M. Computationally efficient confidence intervals for cross-validated area under the ROC curve estimates. *Electron J Stat* 2015;**9**:1583–607.

31. Rosenbaum PR. Model-Based Direct Adjustment. *J Am Stat Assoc* 1987;**82**:387–94.

32. van der Laan MJ, Rubin D. Targeted Maximum Likelihood Learning. *Int J Biostat* 2006;**2**. doi:10.2202/1557-4679.1043

33. Porter KE, Gruber S, van der Laan MJ, *et al.* The relative performance of targeted maximum likelihood estimators. *Int J Biostat* 2011;**7**. doi:10.2202/1557-4679.1308

34. Wollenstein-Betech S, Cassandras CG, Paschalidis IC. Personalized predictive models for symptomatic COVID-19 patients using basic preconditions: Hospitalizations, mortality, and the need for an ICU or ventilator. *Int J Med Inform* 2020;**142**:104258.

35. Parra-Bracamonte GM, Lopez-Villalobos N, Parra-Bracamonte FE. Clinical characteristics and risk factors for mortality of patients with COVID-19 in a large data set from Mexico. *Ann Epidemiol* 2020;**52**:93–8.e2.

36. Hernández-Galdamez DR, González-Block MÁ, Romo-Dueñas DK, *et al.* Increased Risk of Hospitalization and Death in Patients with COVID-19 and Pre-existing Noncommunicable Diseases and Modifiable Risk Factors in Mexico. *Arch Med Res* 2020;**51**:683–9.

For peer review only

Fig. 1. Mortality risk prediction comparing age only logistic regression and super learner

GAM: generalized additive model
The smoothed true mortality risk curve is generated using a GAM with integrated smoothness estimation fitted with cubic splines.

For peer review only

Fig. 2. Super learner predicted mortality risk averaged by specific age in two subgroups: those having all obesity, diabetes, and hypertension pre-existing conditions versus those without

For peer review only

For peer review only - http://bmjopen.bmj.com/site/about/guidelines.xhtml

Supplemental Material

SupplementalMaterial.pdf:

Table S1. TableS1 – [Complete table of baseline variables and pre-existing conditions]

Table S2. TableS2 – [Weighted combination of the super learner fit]

Table S3. TableS3 – [Top 5 ranked most important variables for prediction]

Table S4. TableS4 – [Targeted maximum likelihood estimation adjusted mortality risk, with or without the pre-existing condition]

Figure S1. FigS1 – [Flowchart for analytic sample development]

Figure S2. FigS2 – [Age distribution for laboratory-confirmed COVID-19 patients]

Figure S3. FigS3 – [Prevalence of pre-existing conditions prevalence over time]

Figure S4. FigS4 – [Prediction variable importance predicted using the super learner fit]

Figure S5. FigS5 – [Relative risk for each pre-existing condition associated with mortality]

For peer review only

For peer review only - http://bmjopen.bmj.com/site/about/guidelines.xhtml

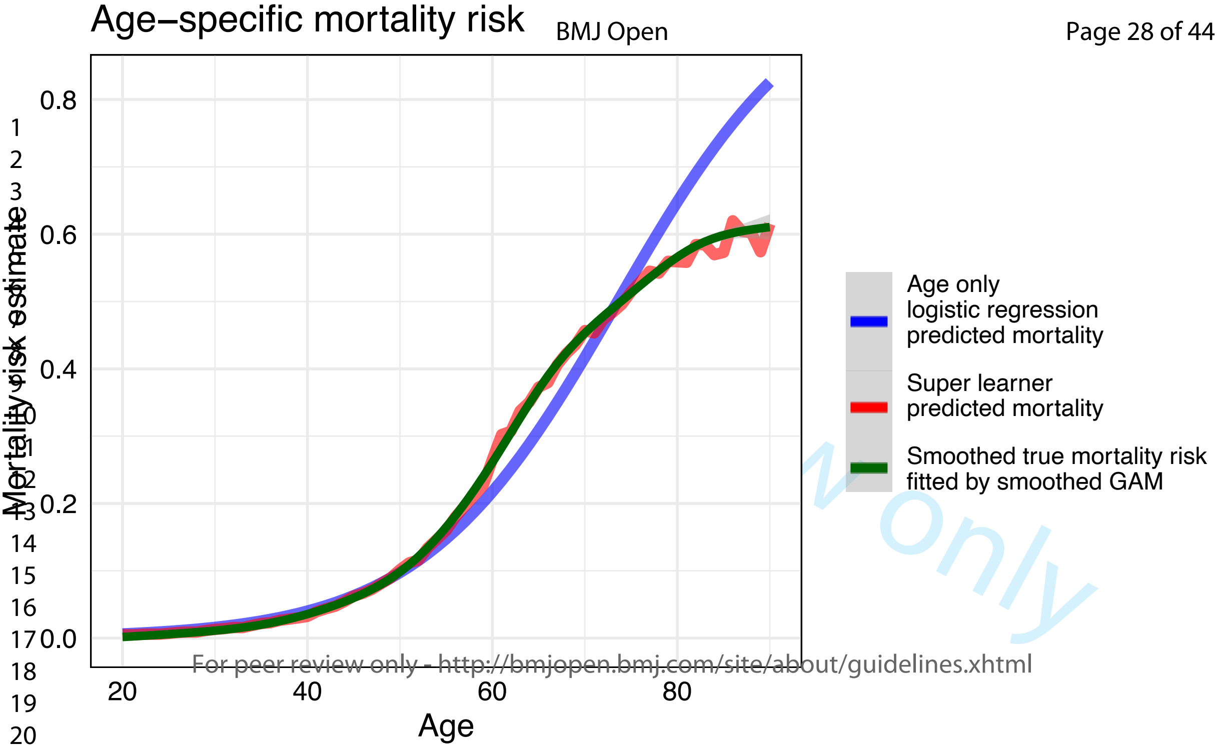

For peer review only - http://bmjopen.bmj.com/site/about/guidelines.xhtml

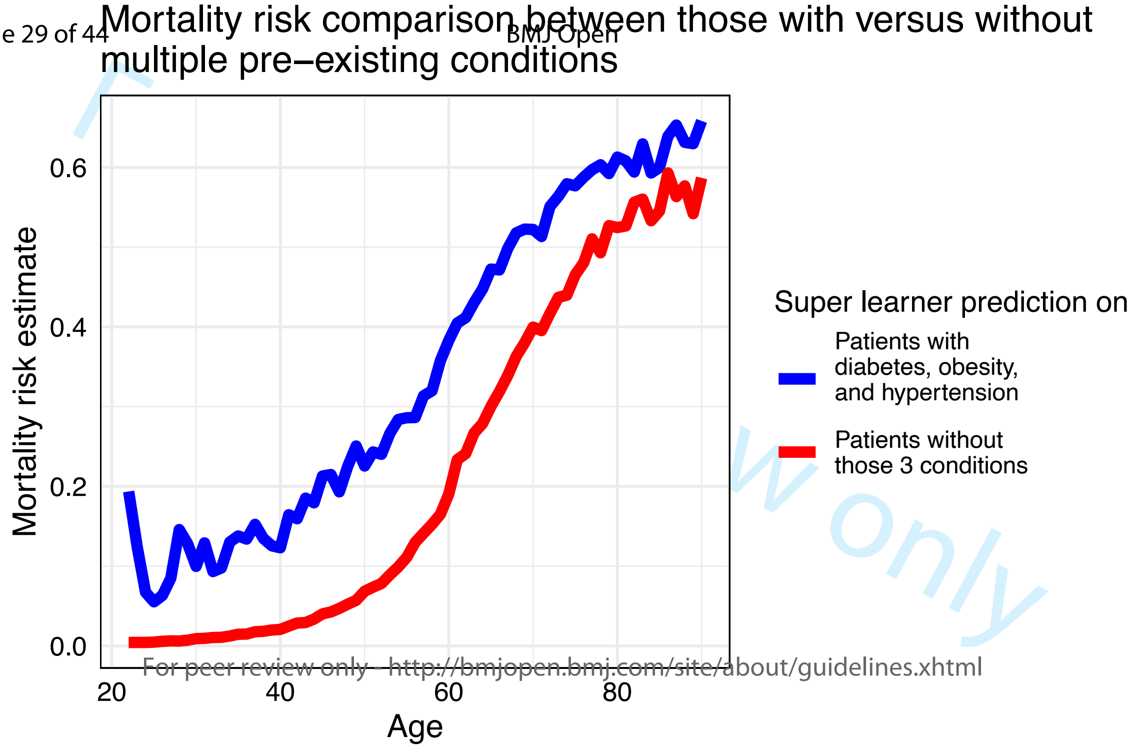

For peer review only - http://bmjopen.bmj.com/site/about/guidelines.xhtml

Supplemental Material

Who is most at risk of dying if infected with SARS-CoV-2? A mortality risk factor analysis using machine learning of COVID-19 patients over time: a large population-based cohort study in Mexico.

Lauren D. Liao,* Alan E. Hubbard, Juan Pablo Gutiérrez, Arturo Juárez-Flores, Kendall Kikkawa, Ronit Gupta, Yana Yarmolich, Iván de Jesús Ascencio-Montiel, Stefano M. Bertozzi

For peer review only

For peer review only - http://bmjopen.bmj.com/site/about/guidelines.xhtml

Table of Contents

Table S1. Complete table of baseline variables and pre-existing conditions 3

Table S2. Weighted combination of the super learner fit 7

Table S3. Top 5 ranked most important variables for prediction 8

Table S4. Targeted maximum likelihood estimation adjusted mortality risk, with or without the pre-existing condition 9

Fig. S1. Flowchart for analytic sample development 10

Fig. S2. Age distribution for laboratory-confirmed COVID-19 patients 11

Fig. S3. Prevalence of pre-existing conditions prevalence over time 12

Fig. S4. Prediction variable importance predicted using the super learner fit 13

Fig. S5. Relative risk for each pre-existing condition associated with mortality 14

For peer review only

For peer review only - http://bmjopen.bmj.com/site/about/guidelines.xhtml

Table S1. Complete table of baseline variables and pre-existing conditions

	All time (2020/03-2021/11)	Phase 1 (2020/03-2020/10)	Phase 2 (2020/11-2021/03)	Phase 3 (2021/04-2021/11)
sample size	1,423,720	303,278	425,698	694,744
Demographic variables				
Age in years (mean (SD))	42.15 (15.70)	46.41 (16.04)	44.89 (16.27)	38.61 (14.34)
Sex = male (%)	729,782 (51.3)	158,248 (52.2)	218,165 (51.2)	353,369 (50.9)
Insured by IMSS = 1(%)	1,358,440 (95.4)	288,588 (95.2)	402,754 (94.6)	667,098 (96.0)
Indigenous = 1 (%)	7,381 (0.5)	2,200 (0.7)	1,628 (0.4)	3,553 (0.5)
Year-month patient initiated care				
2020/03	1,061 (0.1)	1,061 (0.3)	0 (0.0)	0 (0.0)
2020/04	10,832 (0.8)	10,832 (3.6)	0 (0.0)	0 (0.0)
2020/05	30,720 (2.2)	30,720 (10.1)	0 (0.0)	0 (0.0)
2020/06	51,079 (3.6)	51,079 (16.8)	0 (0.0)	0 (0.0)
2020/07	60,780 (4.3)	60,780 (20.0)	0 (0.0)	0 (0.0)
2020/08	49,618 (3.5)	49,618 (16.4)	0 (0.0)	0 (0.0)
2020/09	44,758 (3.1)	44,758 (14.8)	0 (0.0)	0 (0.0)
2020/10	54,430 (3.8)	54,430 (17.9)	0 (0.0)	0 (0.0)
2020/11	65,437 (4.6)	0 (0.0)	65,437 (15.4)	0 (0.0)
2020/12	93,748 (6.6)	0 (0.0)	93,748 (22.0)	0 (0.0)
2021/01	145,858 (10.2)	0 (0.0)	145,858 (34.3)	0 (0.0)
2021/02	68,421 (4.8)	0 (0.0)	68,421 (16.1)	0 (0.0)
2021/03	52,234 (3.7)	0 (0.0)	52,234 (12.3)	0 (0.0)
2021/04	35,181 (2.5)	0 (0.0)	0 (0.0)	35,181 (5.1)

For peer review only

2021/05	26,300 (1.8)	0 (0.0)	0 (0.0)	26,300 (3.8)
2021/06	45,986 (3.2)	0 (0.0)	0 (0.0)	45,986 (6.6)
2021/07	170,212 (12.0)	0 (0.0)	0 (0.0)	170,212 (24.5)
2021/08	249,477 (17.5)	0 (0.0)	0 (0.0)	249,477 (35.9)
2021/09	116,569 (8.2)	0 (0.0)	0 (0.0)	116,569 (16.8)
2021/10	48,515 (3.4)	0 (0.0)	0 (0.0)	48,515 (7.0)
2021/11	2,504 (0.2)	0 (0.0)	0 (0.0)	2,504 (0.4)
Mexican states (%)				
Aguascalientes	26,420 (1.9)	6,897 (2.3)	12,350 (2.9)	7,173 (1.0)
Baja California	43,925 (3.1)	13,677 (4.5)	14,188 (3.3)	16,060 (2.3)
Baja California Sur	24,521 (1.7)	4,300 (1.4)	5,423 (1.3)	14,798 (2.1)
Campeche	9,557 (0.7)	1,728 (0.6)	765 (0.2)	7,064 (1.0)
CDMX 1 Noroeste	32,552 (2.3)	5,374 (1.8)	13,174 (3.1)	14,004 (2.0)
CDMX 2 Noreste	54,249 (3.8)	11,370 (3.7)	20,273 (4.8)	22,606 (3.3)
CDMX 3 Suroeste	42,896 (3.0)	9,701 (3.2)	17,588 (4.1)	15,607 (2.2)
CDMX 4 Sureste	62,097 (4.4)	13,248 (4.4)	25,899 (6.1)	22,950 (3.3)
Chiapas	14,826 (1.0)	2,836 (0.9)	1,801 (0.4)	10,189 (1.5)
Chihuahua	23,229 (1.6)	6,489 (2.1)	6,879 (1.6)	9,861 (1.4)
Coahuila	48,933 (3.4)	16,355 (5.4)	15,459 (3.6)	17,119 (2.5)
Colima	18,310 (1.3)	2,997 (1.0)	2,633 (0.6)	12,680 (1.8)
Durango	19,738 (1.4)	6,228 (2.1)	6,674 (1.6)	6,836 (1.0)
Guanajuato	61,570 (4.3)	11,595 (3.8)	29,274 (6.9)	20,701 (3.0)
Guerrero	23,871 (1.7)	4,502 (1.5)	3,920 (0.9)	15,449 (2.2)
Hidalgo	23,673 (1.7)	4,658 (1.5)	8,266 (1.9)	10,749 (1.5)
Jalisco	104,054 (7.3)	19,491 (6.4)	27,868 (6.5)	56,695 (8.2)

For peer review only - http://bmjopen.bmj.com/site/about/guidelines.xhtml

Mexico Oriente	108,067 (7.6)	20,368 (6.7)	39,633 (9.3)	48,066 (6.9)
Mexico Poniente	48,973 (3.4)	11,632 (3.8)	17,026 (4.0)	20,315 (2.9)
Michoacan	30,570 (2.1)	6,246 (2.1)	7,221 (1.7)	17,103 (2.5)
Morelos	18,797 (1.3)	2,845 (0.9)	6,817 (1.6)	9,135 (1.3)
Nayarit	23,934 (1.7)	3,378 (1.1)	2,994 (0.7)	17,562 (2.5)
Nuevo Leon	105,912 (7.4)	23,776 (7.8)	30,114 (7.1)	52,022 (7.5)
Oaxaca	24,324 (1.7)	4,493 (1.5)	5,391 (1.3)	14,440 (2.1)
Puebla	50,998 (3.6)	9,287 (3.1)	17,932 (4.2)	23,779 (3.4)
Queretaro	41,977 (2.9)	4,707 (1.6)	19,259 (4.5)	18,011 (2.6)
Quintana Roo	38,390 (2.7)	4,607 (1.5)	4,542 (1.1)	29,241 (4.2)
San Luis Potosi	26,118 (1.8)	7,353 (2.4)	7,573 (1.8)	11,192 (1.6)
Sinaloa	44,333 (3.1)	11,030 (3.6)	9,405 (2.2)	23,898 (3.4)
Sonora	27,691 (1.9)	7,245 (2.4)	6,083 (1.4)	14,363 (2.1)
Tabasco	16,004 (1.1)	2,622 (0.9)	1,719 (0.4)	11,663 (1.7)
Tamaulipas	38,941 (2.7)	8,504 (2.8)	7,442 (1.7)	22,995 (3.3)
Tlaxcala	13,809 (1.0)	2,769 (0.9)	4,957 (1.2)	6,083 (0.9)
Veracruz Norte	41,804 (2.9)	10,002 (3.3)	6,801 (1.6)	25,001 (3.6)
Veracruz Sur	35,019 (2.5)	10,292 (3.4)	4,970 (1.2)	19,757 (2.8)
Yucatan	35,126 (2.5)	5,968 (2.0)	5,091 (1.2)	24,067 (3.5)
Zacatecas	18,512 (1.3)	4,708 (1.6)	8,294 (1.9)	5,510 (0.8)
Pre-existing conditions				
Asthma = yes (%)	25,297 (1.8)	7,951 (2.6)	7,765 (1.8)	9,581 (1.4)
Cardiovascular disease = yes (%)	17,816 (1.3)	6,643 (2.2)	6,389 (1.5)	4,784 (0.7)
Chronic liver disease = yes (%)	1,875 (0.1)	710 (0.2)	668 (0.2)	497 (0.1)
COPD = yes (%)	15,390 (1.1)	5,825 (1.9)	5,496 (1.3)	4,069 (0.6)

For peer review only - http://bmjopen.bmj.com/site/about/guidelines.xhtml

Diabetes = yes (%)	169,869 (11.9)	55,551 (18.3)	61,120 (14.4)	53,198 (7.7)
Hemolytic anemia = yes (%)	705 (0.0)	276 (0.1)	246 (0.1)	183 (0.0)
HIV = yes (%)	4,717 (0.3)	1,133 (0.4)	1,425 (0.3)	2,159 (0.3)
Hypertension = yes (%)	228,901 (16.1)	72,615 (23.9)	83,735 (19.7)	72,551 (10.4)
Immunosuppression = yes (%)	10,434 (0.7)	4,102 (1.4)	3,453 (0.8)	2,879 (0.4)
Neurological disease = yes (%)	1,645 (0.1)	544 (0.2)	559 (0.1)	542 (0.1)
Obesity = yes (%)	181,736 (12.8)	55,965 (18.5)	60,217 (14.1)	65,554 (9.4)
Smoking = yes (%)	87,161 (6.1)	21,253 (7.0)	28,346 (6.7)	37,562 (5.4)
Cancer diagnosis = yes (%)	3,751 (0.3)	1,178 (0.4)	1,317 (0.3)	1,256 (0.2)
Renal disease diagnosis = yes (%)	24,099 (1.7)	8,912 (2.9)	8,555 (2.0)	6,632 (1.0)
Tuberculosis = yes (%)	675 (0.0)	203 (0.1)	218 (0.1)	254 (0.0)

CDMX: Ciudad de México; COPD: Chronic obstructive pulmonary disease; HIV: human immunodeficiency virus; SD: standard deviation
Mexican states refer to where the patient was treated

For peer review only

Table S2. Weighted combination of the super learner fit

Machine learning candidate algorithm	Weights	Mean squared error	Standard error
Bayesian additive regression trees	0	0.266	0.0002
Bayesian generalized linear model	0	0.067	0.0003
Elastic net regression	0	0.068	0.0004
Empirical mean	0	0.094	0.0005
XGBoost (multiple tuning)	0.596 (combined)	0.065 (on average)	0.0003 (on average)
Generalized additive model	0.222	0.066	0.0004
LASSO regression	0	0.067	0.0004
Logistic regression	0	0.067	0.0004
Multivariate Adaptive Regression Splines	0	0.068	0.0004
Random forest	0.181	0.066	0.0002
Ridge regression	0	0.067	0.0002

LASSO: least absolute shrinkage and selection operator; XGBoost: extreme gradient boosting
XGBoost coefficients are combined; mean squared error and standard error were averaged.

For peer review only - http://bmjopen.bmj.com/site/about/guidelines.xhtml

Table S3. Top 5 ranked most important variables for prediction

	All time (2020/03-2021/11)	Phase 1 (2020/03-2020/10)	Phase 2 (2020/11-2021/03)	Phase 3 (2021/04-2021/11)
Rank 1	Age 0.147	Age 0.209	Age 0.208	Age 0.069
Rank 2	Year-month patient initiated care 0.014	Renal disease 0.008	Mexican state 0.007	Renal disease 0.004
Rank 3	Renal disease 0.005	Sex 0.007	Renal disease 0.006	Mexican state 0.003
Rank 4	Mexican state 0.004	Year-month patient initiated care 0.007	Insured by IMSS 0.006	Diabetes 0.003
Rank 5	Sex 0.004	Mexican state 0.006	Sex 0.006	Insured by IMSS 0.002

IMSS: Mexican Institute of Social Security
Mexican state refers to where the patient was treated.
Measured by the log-likelihood difference in prediction pre-post permutation of each variable while holding all others constant.

For peer review only

Table S4. Targeted maximum likelihood estimation adjusted mortality risk, with or without the pre-existing condition

	All time (2020/03-2021/11)		Phase 1 (2020/03-2020/10)		Phase 2 (2020/11-2021/03)		Phase 3 (2021/04-2021/11)	
	with	without	with	without	with	without	with	without
Renal disease	0.381	0.101	0.439	0.170	0.425	0.142	0.305	0.046
Diabetes	0.173	0.094	0.247	0.161	0.214	0.135	0.104	0.041
Hypertension	0.162	0.093	0.231	0.162	0.201	0.134	0.097	0.041
Obesity	0.141	0.099	0.212	0.168	0.177	0.141	0.080	0.045
Smoking	0.110	0.105	0.176	0.176	0.146	0.147	0.056	0.048
Asthma	0.109	0.105	0.166	0.177	0.139	0.147	0.059	0.049

For peer review only

For peer review only - http://bmjopen.bmj.com/site/about/guidelines.xhtml

Fig. S1. Flowchart for analytic sample development

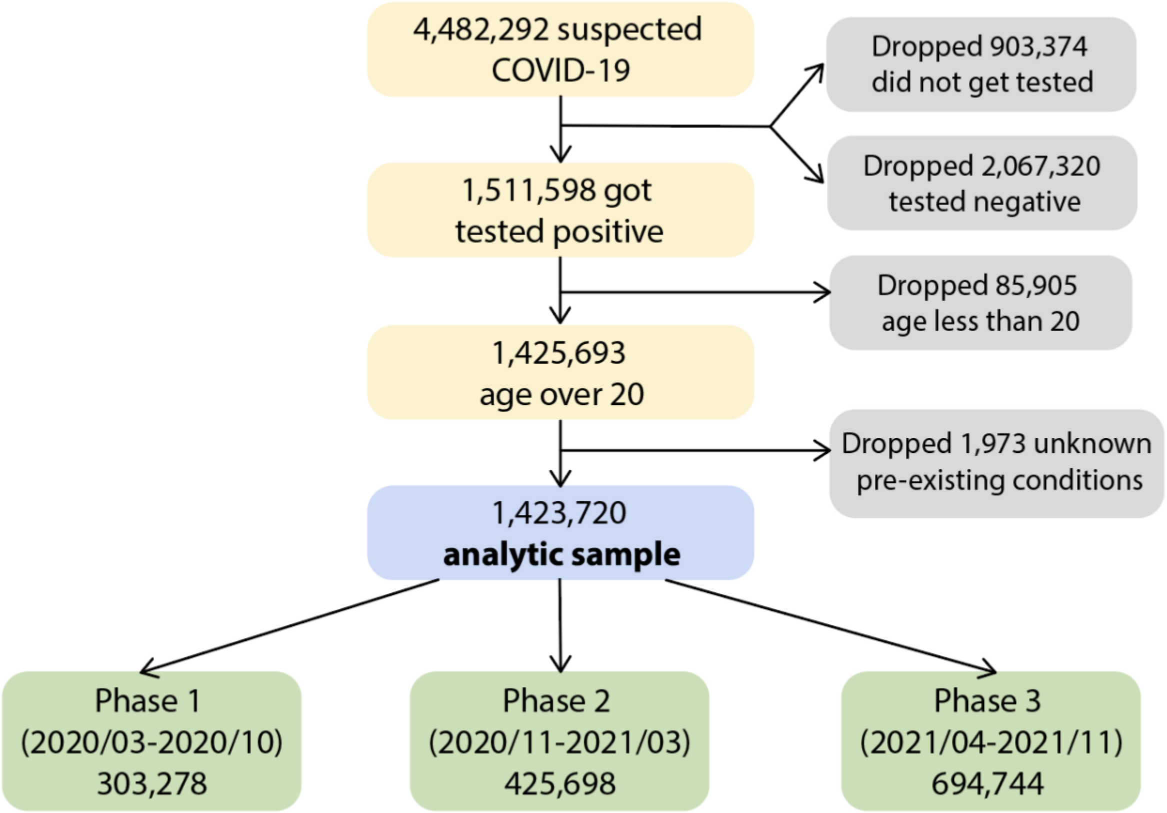

For peer review only - http://bmjopen.bmj.com/site/about/guidelines.xhtml

Fig. S2. Age distribution for laboratory-confirmed COVID-19 patients

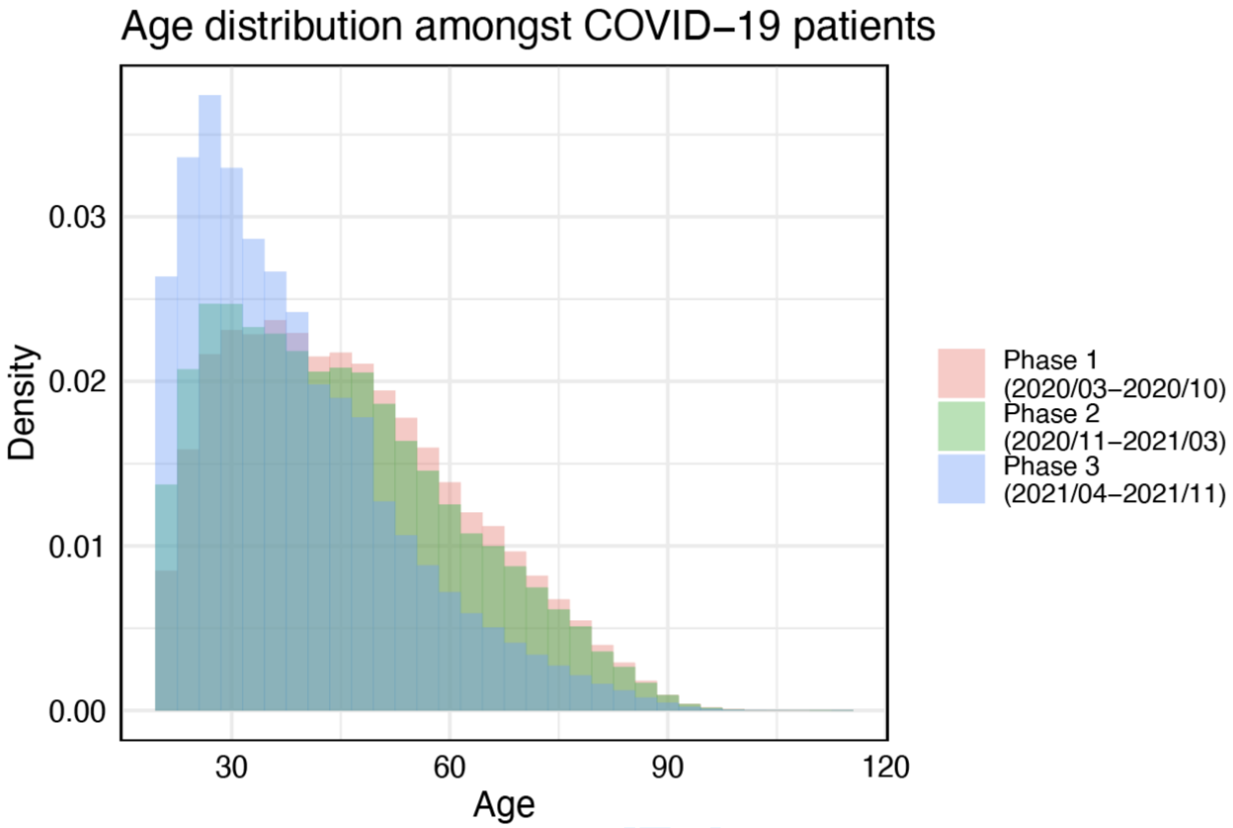

For peer review only - http://bmjopen.bmj.com/site/about/guidelines.xhtml

Fig. S3. Prevalence of pre-existing conditions prevalence over time

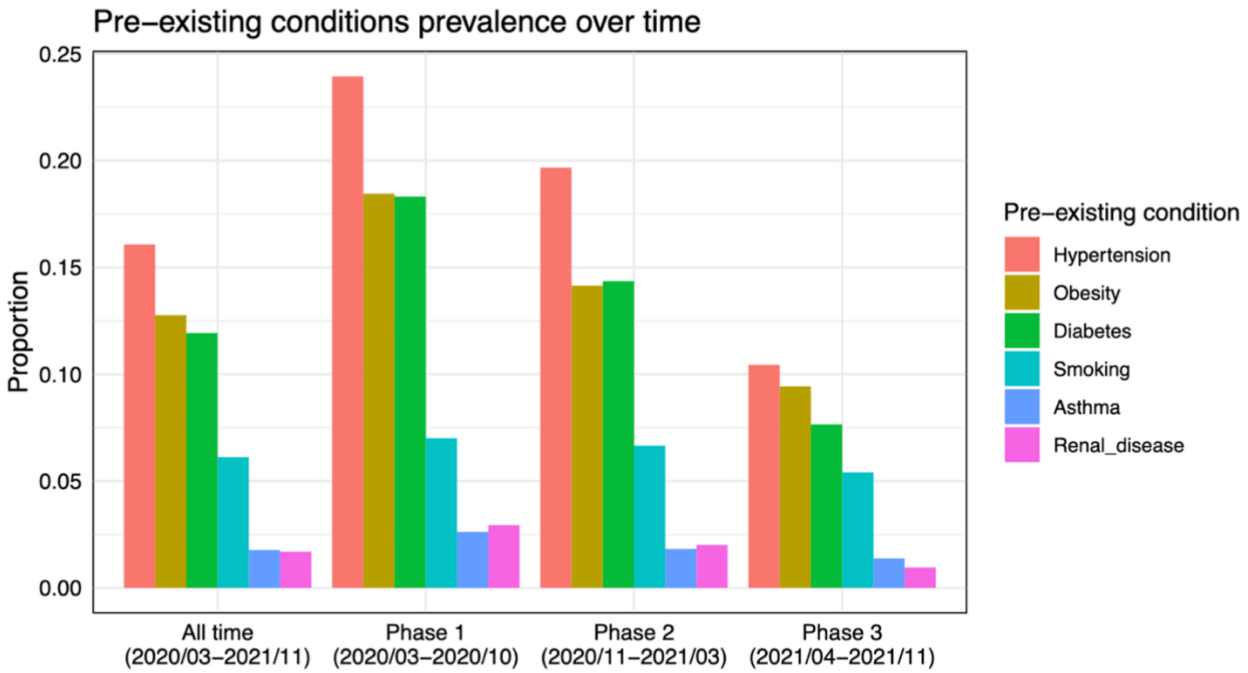

For peer review only - http://bmjopen.bmj.com/site/about/guidelines.xhtml

Fig. S4. Prediction variable importance predicted using the super learner fit

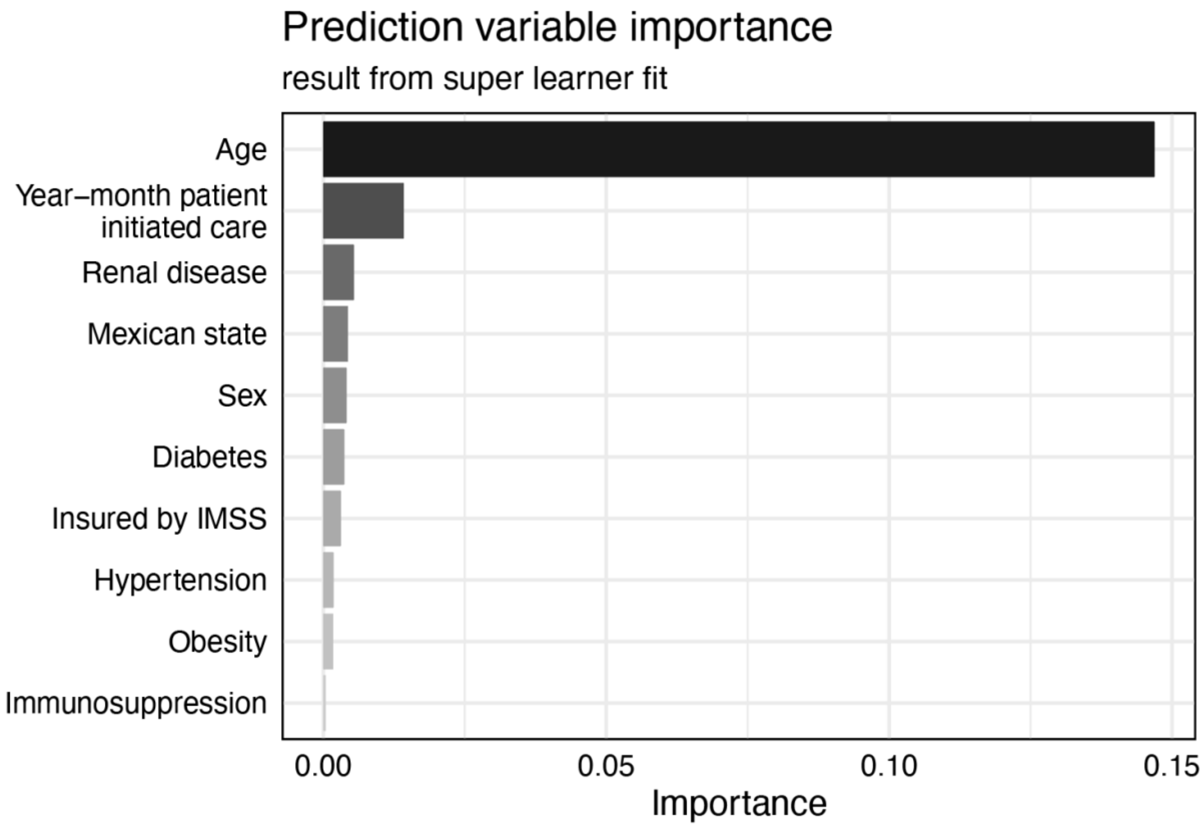

IMSS: Mexican Institute of Social Security

For peer review only - http://bmjopen.bmj.com/site/about/guidelines.xhtml

Fig. S5. Relative risk for each pre-existing condition associated with mortality

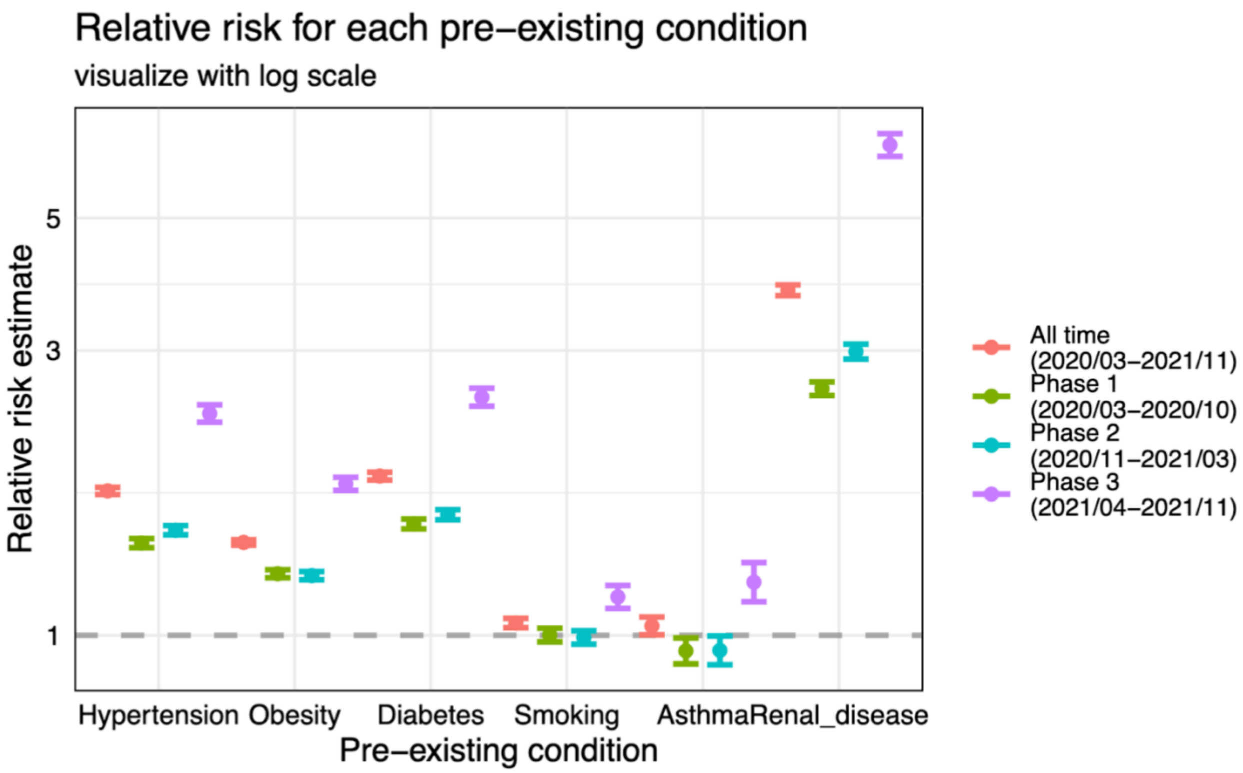

Error bars reflect a 95% confidence interval.

For peer review only - http://bmjopen.bmj.com/site/about/guidelines.xhtml

STROBE Statement—Checklist of items that should be included in reports of ***cohort studies***

	Item No	Recommendation	Page No
**Title and abstract**	1	(*a*) Indicate the study's design with a commonly used term in the title or the abstract	1
		(*b*) Provide in the abstract an informative and balanced summary of what was done and what was found	2-3
**Introduction**			
Background/rationale	2	Explain the scientific background and rationale for the investigation being reported	5-7
Objectives	3	State specific objectives, including any prespecified hypotheses	7
**Methods**			
Study design	4	Present key elements of study design early in the paper	7-8
Setting	5	Describe the setting, locations, and relevant dates, including periods of recruitment, exposure, follow-up, and data collection	7-8
Participants	6	(*a*) Give the eligibility criteria, and the sources and methods of selection of participants. Describe methods of follow-up	7-8
		(*b*) For matched studies, give matching criteria and number of exposed and unexposed	N/A
Variables	7	Clearly define all outcomes, exposures, predictors, potential confounders, and effect modifiers. Give diagnostic criteria, if applicable	8-10
Data sources/ measurement	8*	For each variable of interest, give sources of data and details of methods of assessment (measurement). Describe comparability of assessment methods if there is more than one group	8
Bias	9	Describe any efforts to address potential sources of bias	16
Study size	10	Explain how the study size was arrived at	9-10
Quantitative variables	11	Explain how quantitative variables were handled in the analyses. If applicable, describe which groupings were chosen and why	8-9
Statistical methods	12	(*a*) Describe all statistical methods, including those used to control for confounding	10-12
		(*b*) Describe any methods used to examine subgroups and interactions	9-10
		(*c*) Explain how missing data were addressed	9
		(*d*) If applicable, explain how loss to follow-up was addressed	N/A
		(*e*) Describe any sensitivity analyses	N/A
**Results**			
Participants	13*	(a) Report numbers of individuals at each stage of study—eg numbers potentially eligible, examined for eligibility, confirmed eligible, included in the study, completing follow-up, and analysed	9-10
		(b) Give reasons for non-participation at each stage	9
		(c) Consider use of a flow diagram	9
Descriptive data	14*	(a) Give characteristics of study participants (eg demographic, clinical, social) and information on exposures and potential confounders	8-9
		(b) Indicate number of participants with missing data for each variable of interest	N/A
		(c) Summarise follow-up time (eg, average and total amount)	8-9
Outcome data	15*	Report numbers of outcome events or summary measures over time	9

For peer review only - http://bmjopen.bmj.com/site/about/guidelines.xhtml

Main results	16	(*a*) Give unadjusted estimates and, if applicable, confounder-adjusted estimates and their precision (eg, 95% confidence interval). Make clear which confounders were adjusted for and why they were included	13
		(*b*) Report category boundaries when continuous variables were categorized	N/A
		(*c*) If relevant, consider translating estimates of relative risk into absolute risk for a meaningful time period	14
Other analyses	17	Report other analyses done—eg analyses of subgroups and interactions, and sensitivity analyses	13-14
**Discussion**			
Key results	18	Summarise key results with reference to study objectives	15
Limitations	19	Discuss limitations of the study, taking into account sources of potential bias or imprecision. Discuss both direction and magnitude of any potential bias	16-17
Interpretation	20	Give a cautious overall interpretation of results considering objectives, limitations, multiplicity of analyses, results from similar studies, and other relevant evidence	16
Generalisability	21	Discuss the generalisability (external validity) of the study results	16-17
**Other information**			
Funding	22	Give the source of funding and the role of the funders for the present study and, if applicable, for the original study on which the present article is based	19

*Give information separately for exposed and unexposed groups.

Note: An Explanation and Elaboration article discusses each checklist item and gives methodological background and published examples of transparent reporting. The STROBE checklist is best used in conjunction with this article (freely available on the Web sites of PLoS Medicine at http://www.plosmedicine.org/, Annals of Internal Medicine at http://www.annals.org/, and Epidemiology at http://www.epidem.com/). Information on the STROBE Initiative is available at http://www.strobe-statement.org.

For peer review only

For peer review only - http://bmjopen.bmj.com/site/about/guidelines.xhtml

BMJ Open

Who is most at risk of dying if infected with SARS-CoV-2? A mortality risk factor analysis using machine learning of COVID-19 patients over time: a large population-based cohort study in Mexico.

Journal:	*BMJ Open*
Manuscript ID	bmjopen-2023-072436.R1
Article Type:	Original research
Date Submitted by the Author:	29-Aug-2023

Complete List of Authors:	Liao, Lauren; University of California Berkeley, Division of Biostatistics<br>Hubbard, Alan; University of California Berkeley, Division of Biostatistics<br>Gutierrez, Juan Pablo; Universidad Nacional Autónoma de México, Center for Policy, Population & Health Research, School of Medicine<br>Juárez, Arturo; Universidad Nacional Autónoma de México<br>Kikkawa, Kendall; Micron Technology<br>Gupta, Ronit; University of California Berkeley<br>Yarmolich, Yana; University of California Berkeley<br>de Jesús Ascencio-Montiel, Iván; Instituto Mexicano del Seguro Social<br>Bertozzi, Stefano; University of California Berkeley School of Public Health; University of Washington School of Public Health

**Primary Subject Heading**:	Epidemiology
Secondary Subject Heading:	Public health
Keywords:	COVID-19, STATISTICS & RESEARCH METHODS, Risk Factors, Hypertension < CARDIOLOGY, General diabetes < DIABETES & ENDOCRINOLOGY, Epidemiology < INFECTIOUS DISEASES

SCHOLARONE™
Manuscripts

I, the Submitting Author has the right to grant and does grant on behalf of all authors of the Work (as defined in the below author licence), an exclusive licence and/or a non-exclusive licence for contributions from authors who are: i) UK Crown employees; ii) where BMJ has agreed a CC-BY licence shall apply, and/or iii) in accordance with the terms applicable for US Federal Government officers or employees acting as part of their official duties; on a worldwide, perpetual, irrevocable, royalty-free basis to BMJ Publishing Group Ltd ("BMJ") its licensees and where the relevant Journal is co-owned by BMJ to the co-owners of the Journal, to publish the Work in this journal and any other BMJ products and to exploit all rights, as set out in our licence.

The Submitting Author accepts and understands that any supply made under these terms is made by BMJ to the Submitting Author unless you are acting as an employee on behalf of your employer or a postgraduate student of an affiliated institution which is paying any applicable article publishing charge ("APC") for Open Access articles. Where the Submitting Author wishes to make the Work available on an Open Access basis (and intends to pay the relevant APC), the terms of reuse of such Open Access shall be governed by a Creative Commons licence – details of these licences and which Creative Commons licence will apply to this Work are set out in our licence referred to above.

Other than as permitted in any relevant BMJ Author's Self Archiving Policies, I confirm this Work has not been accepted for publication elsewhere, is not being considered for publication elsewhere and does not duplicate material already published. I confirm all authors consent to publication of this Work and authorise the granting of this licence.

For peer review only

Who is most at risk of dying if infected with SARS-CoV-2? A mortality risk factor analysis using machine learning of COVID-19 patients over time: a large population-based cohort study in Mexico.

Lauren D. Liao,[1*] Alan E. Hubbard,[1] Juan Pablo Gutiérrez,[2] Arturo Juárez-Flores,[2] Kendall Kikkawa,[3] Ronit Gupta,[1] Yana Yarmolich,[1] Iván de Jesús Ascencio-Montiel,[4] Stefano M. Bertozzi[1,5,6]

Affiliations:

[1] University of California, Berkeley, School of Public Health, Berkeley, CA, USA

[2] Center for Policy, Population & Health Research, School of Medicine, Universidad Nacional Autónoma de México, Mexico City, Mexico

[3] Micron Technology, Boise, ID, USA

[4] Instituto Mexicano del Seguro Social, CDMX, México

[5] University of Washington School of Public Health, Seattle, WA, USA

[6] Instituto Nacional de Salud Pública, Cuernavaca, MOR, México

***Correspondence to:** ldliao@berkeley.edu (LDL)

Word count: 3054

For peer review only - http://bmjopen.bmj.com/site/about/guidelines.xhtml

Abstract

Objective: COVID-19 would kill fewer people if health programs can predict who is at higher risk of mortality because resources can be targeted to protect those people from infection. We predict mortality in a very large population in Mexico with machine learning using demographic variables and pre-existing conditions.

Design Cohort study.

Setting March 2020 to November 2021 in Mexico, nationally represented.

Participants 1.4 million laboratory-confirmed COVID-19 patients in Mexico at or over 20 years of age.

Primary and secondary outcome measures Analysis is performed on data from March 2020 to November 2021 and over three phases: (1) from March to October in 2020, (2) from November 2020 to March 2021, and (3) from April to November 2021. We predict mortality using an ensemble machine learning method, super learner, and independently estimate the adjusted mortality relative risk of each pre-existing condition using targeted maximum likelihood estimation.

Results Super learner fit has a high predictive performance (C-statistic: 0.907), where age is the most predictive factor for mortality. After adjusting for demographic factors, renal disease, hypertension, diabetes, and obesity are the most impactful pre-existing conditions. Phase analysis shows that the adjusted mortality risk decreased over time while relative risk increased for each pre-existing condition.

Conclusions While age is the most important predictor of mortality, younger individuals with hypertension, diabetes and obesity are at comparable mortality risk as individuals who are 20

years older without any of the three conditions. Our model can be continuously updated to identify individuals who should most be protected against infection as the pandemic evolves.

Keywords: COVID-19; Epidemiology; Diabetes; Hypertension; Statistical methods; Risk Factors

For peer review only

Strengths and limitations of this study

- Compared to previous studies showing age and the presence of pre-existing conditions as important predictors of mortality, our study leveraged more powerful statistical approaches by examining mortality risk in a very large population (> 4.5 M).
- Analyses based on a combination of machine learning and causal inference methods for prediction and estimation of risk factor impacts that is robust to model misspecification.
- The phase analyses presented in this study demonstrates marked changes over time in the degree to which different risk factors predict mortality.
- Preexisting conditions are self-reported, and thus subject to potential misspecification.
- Those who are included in the data may not be representative of the socioeconomic status of the entire population; thus, extrapolation of the result should be done with caution.

For peer review only

For peer review only - http://bmjopen.bmj.com/site/about/guidelines.xhtml

Introduction

The probability of mortality associated with SARS-CoV-2 infection has varied enormously over time, among countries, and among population groups within countries [1]. Interest in understanding who is at a higher risk of death has grown as this heterogeneity became more apparent. Identifying people at higher risk of severe disease and death will help health systems better respond and focus prevention resources on protecting them. We examine Mexico, a country with a very high reported case-fatality rate (4.7%) among those who have laboratory-confirmed coronavirus disease 2019 (COVID-19) as of September 23, 2022 [2].

Previous analyses in Mexico have found diabetes, obesity, hypertension, immunosuppression, and renal disease to be significant risk factors along with age and sex. Multiple authors have identified obesity and diabetes as important risk factors for mortality [3–6]. Escobedo de-la Peña et al. also found a strong association with hypertension, which is consistent with results from Giannouchos et al. [6, 7]. Late-stage chronic kidney disease, although less prevalent, has also consistently been identified as a COVID-19 mortality risk factor. Older/ male patients tend to have higher mortality risks than younger/ female patients [4, 6, 7]. In a previous analysis, we found interactions between those comorbidities, suggesting a synergic effect when having more than one of diabetes, hypertension, and obesity (larger odds ratio when reporting the 3 conditions vs. one or two) [8]. We also found that the odds ratio increased by age group with those over age 80 having 30-fold the risk of those 20 to 29 [8]. One important consideration is that the prevalence of diabetes and hypertension is positively associated with age, so it has not been clear how this interaction is related to mortality risk. A more adaptive analysis performed by Martínez-Martínez et al. developed a prediction model for severity of COVID-19, defined by hospitalization and/or mortality. They examined the

For peer review only - http://bmjopen.bmj.com/site/about/guidelines.xhtml

relationship of 14 variables with hospitalization and mortality using interaction terms and splines to account for non-linear relationships [9].

The pattern of age, sex, and comorbidities being associated with higher mortality risk is not specific to Mexico, and the global literature on such associations is extensive. Researchers have identified old age, diabetes, obesity, chronic renal failure, and congestive heart failure to be strongly associated with severe infection amongst both sexes in the Spanish population [10]. Researchers in Brazil showed that older age, male, kidney disease, obesity and/ or diabetes are strong predictors of mortality amongst other comorbidities such as chronic liver disease, immunosuppression, and cardiovascular disease [11, 12]. Another study used United Kingdom Biobank data and showed that pre-existing dementia, diabetes, chronic obstructive pulmonary disease (COPD), pneumonia, and depression were positively associated with risk of hospitalization and death [13]. An analysis from France found age, diabetes, hypertension, obesity, cancer, and kidney and lung transplants to be associated with risk of COVID-19-related hospitalization and mortality, among others [14]. A Canadian study reported dementia, chronic kidney disease, cardiovascular disease, diabetes, COPD, severe mental illness, organ transplant, hypertension, and cancer to be significant predictors of mortality [15]. Studies presented here is a non-exhaustive list of research studying COVID-19 risk factors and mortality. Recent meta-analyses and systematic reviews find significant mortality attributed to these pre-existing conditions [1, 16-18]. Our goal in this study is not only to predict mortality using demographic factors and comorbidities, but to show how those predictions change over time in this rapidly evolving pandemic.

Although mortality risk estimation and risk factor identification have been examined in prior studies, we are concerned about the statistical validity and interpretation of the standard

For peer review only - http://bmjopen.bmj.com/site/about/guidelines.xhtml

methods. A commonly used prediction tool, logistic regression, assumes a linear relationship of predictors against the log odds of mortality risk, but this logit-linear assumption will lead inevitably to biased estimates of risk (either under- or over-predict the risk) for subsets of the population. We instead used flexible, data-adaptive methods that can capture non-linearities in the dose-response, such as potential nonlinear interactions between the predictors (e.g., the potential interaction of age and diabetes on predicting death) [19, 20]. The better the model fits the study population; the more likely estimates are closer to the true joint relationship of mortality and risk factors.

We included pre-existing conditions, demographic variables, the Mexican state where the patient was treated, and the month that the patient initiated care to fit our prediction algorithm. We conducted the analysis using an ensemble machine learning algorithm, super learner, to form optimal combination of predictions from multiple machine learning methods [19, 20]. We also estimated the comparative importance of variables for mortality risk prediction (holding all other variables constant) by nonparametrically estimating quantities inspired by causal parameters (parameters that compare so-called counterfactual distributions, in our case, causal relative risks). The statistical goal is to estimate and provide robust inference for impact estimates of the predictors without the arbitrary modeling assumptions that characterize the great majority of prior work [21].

Methods

Study population and design

The study population is drawn from the Mexican Social Security Institute (IMSS), a vertically integrated insurance and health system that provides coverage for over 60 million

For peer review only - http://bmjopen.bmj.com/site/about/guidelines.xhtml

private sector employees and their families, including their parents, children and spouse. IMSS also provided care as part of the COVID-19 response for some non-beneficiaries, who are also included in the dataset.

The data were recorded from March 1st, 2020, to November 3rd, 2021 in a platform called SINOLAVE. They reflect the entire population of 4,482,292 patients who were registered as receiving care for suspected COVID-19 at an IMSS facility. The dataset and the data entry process have been described previously [22]. The demographic variables include age, sex, insured by IMSS, and indigenous status. The data contains pre-existing conditions reported by the patient or the family at presentation: asthma, cardiovascular disease, chronic liver disease, chronic obstructive pulmonary disease, diabetes, hemolytic anemia, human immunodeficiency virus, hypertension, immunosuppression, neurological disease, obesity, cancer, renal disease and tuberculosis, as well as whether the patient currently smokes. Patients were asked at presentation about their pre-existing health conditions; these were not ascertained with reference to the patient's medical record, even for those patients insured by the IMSS. The data also includes the Mexican state in which the patient received care, COVID-19 test results (from both polymerase chain reaction (PCR) tests and antigen tests), the month that the patient initiated care, and COVID-related mortality. The outcome, death, is ascertained as COVID-related mortality within this study period between March 2020 and November 2021; we only consider deaths after patients initiated care. In addition, we extracted a different dataset from the National Council of Science and Technology to determine the dominant circulating variant in each month [23]. A short summary can be found in **Table 1 (Supplemental Table S1)**. We define COVID-19 positive as a positive PCR or antigen test.

For peer review only - http://bmjopen.bmj.com/site/about/guidelines.xhtml

Table 1. Summary table of baseline variables and pre-existing conditions

	**All time** (2020/03-2021/11)	**Phase 1** (2020/03-2020/10)	**Phase 2** (2020/11-2021/03)	**Phase 3** (2021/04-2021/11)
Sample size	1,423,720	303,278	425,698	694,744
**Demographic variables**				
Age in years (mean (SD))	42.15 (15.70)	46.41 (16.04)	44.89 (16.27)	38.61 (14.34)
Sex = male (%)	729,782 (51.3)	158,248 (52.2)	218,165 (51.2)	353,369 (50.9)
Insured by IMSS = yes (%)	1,358,440 (95.4)	288,588 (95.2)	402,754 (94.6)	667,098 (96.0)
Indigenous = yes (%)	7,381 (0.5)	2,200 (0.7)	1,628 (0.4)	3,553 (0.5)
**Pre-existing conditions**				
Hypertension = yes (%)	228,901 (16.1)	72,615 (23.9)	83,735 (19.7)	72,551 (10.4)
Diabetes = yes (%)	169,869 (11.9)	55,551 (18.3)	61,120 (14.4)	53,198 (7.7)
Obesity = yes (%)	181,736 (12.8)	55,965 (18.5)	60,217 (14.1)	65,554 (9.4)
Smoking = yes (%)	87,161 (6.1)	21,253 (7.0)	28,346 (6.7)	37,562 (5.4)
Asthma = yes (%)	25,297 (1.8)	7,951 (2.6)	7,765 (1.8)	9,581 (1.4)
Renal Disease Diagnosis = yes (%)	24,099 (1.7)	8,912 (2.9)	8,555 (2.0)	6,632 (1.0)
**Outcome**				
Death = yes (%)	149,805 (10.5)	53,530 (17.7)	62,517 (14.7)	33,758 (4.9)

IMSS: Mexican Social Security Institute; SD: standard deviation

For peer review only

From the full data set, we generated an analytic sample (n = 1,423,720) **(Supplemental Figure S1)**. We exclude those under the age of 20 years, those without any positive COVID-19 test result from either the PCR or antigen tests, and those with unknown pre-existing conditions. We also create a phase variable that corresponds to changes in the epidemic curve into three: phase 1 is from March 1st, 2020, to October 31st, 2020, phase 2 is from November 1st, 2020, to

For peer review only - http://bmjopen.bmj.com/site/about/guidelines.xhtml

162 March 31st, 2021, and phase 3 is from April 1st, 2021, to November 3rd, 2021 as previously described [22].

Patient and Public Involvement

Patients or the public were not involved in the design, or conduct, or reporting, or dissemination plans of our research.

Statistical analysis

Mortality risk prediction using super learner (SL)

We predict mortality risks with SL [19, 20], using predictors: pre-existing conditions, demographic variables, the Mexican state where the patient was treated, and the month that the patient initiated care. SL combines a set of user-supplied machine learning algorithms, which includes both simple, parametric fits and flexible algorithms, to create an optimally-weighted combination. This optimal fit is found by creating a combination of algorithms that minimize the cross-validated risk (in our case, the negative log-likelihood). SL has the property that asymptotically it will perform at least as well as the best fitting algorithm in the library [19, 20]. Thus, it is important to include a diverse and large set of learners as candidates to ensure the model can fit complex patterns if warranted, but also, simpler, parametric models if simpler fits are sufficient. The following learners were included in the SL library: Bayesian additive regression trees [24], Bayesian generalized linear model [25], elastic net regression [26], empirical mean, generalized additive model [27], least absolute shrinkage and selection operator regression [28], logistic regression, multivariate adaptive regression splines [29], random forest [30], ridge regression [31], and extreme gradient boosting algorithms [32]. We estimate the prediction performance, via the AUC, and derive a 95% confidence interval for the estimated

For peer review only - http://bmjopen.bmj.com/site/about/guidelines.xhtml

AUC [33]. We compare the SL fit using all predictors listed above to a logistic regression with only age entered as a linear term. We compute the AUC for the resulting SL/logistic regression fits with 3-fold cross validation on the 80%, both on the same data used to estimate SL/logistic regression models (training AUC), as well as a more realistic assessment by using the test set – the left-out 20% of the available data (testing AUC).

To interpret the final prediction model generated by the SL fit, we use the permutation-based variable importance measure to identify variables that influence the SL model's prediction [30]. This is performed by permuting the predictor variables one at a time (keeping the other variables fixed) and measuring the magnitude of the decline on the predictive performance (as measured by the change in the average negative log-likelihood). This provides a list of variables ranked by the relative importance to prediction fit but does not provide information on the variable impact on mortality, which led us to another measure of relative risk (RR) using targeted maximum likelihood estimation (TMLE).

Pre-existing condition relative risk estimate through targeted maximum likelihood estimation

For pre-existing conditions, we estimated a different variable importance measure that is not focused on prediction accuracy but on estimating potential impacts of pre-existing conditions on mortality risk. The impact is estimated by the RR of adjusted means (adjusted for baseline confounders) for the population if everyone had the specific pre-existing condition of interest (the numerator) versus the same population where no one has the specific pre-existing condition (the denominator). To estimate RRs, we used cross-validated targeted minimum-loss-based estimation (cross-validated TMLE). TMLE is a semiparametric, substitution estimator that has

For peer review only - http://bmjopen.bmj.com/site/about/guidelines.xhtml

shown to be asymptotically efficient (unlike the inverse probability of treatment-weighting estimators [34]). It also has some robustness advantages over other semiparametric efficient approaches, such as augmented inverse probability weighting. TMLE estimates parameters that, under certain assumptions, can be interpreted as potential causal impacts of these factors on mortality, in our case, in the form of a causal relative risk. Our ensemble machine learning is optimized for prediction, but it does not directly provide measures of individual variable importance. We augmented our prior SL analysis using the TMLE to generate interpretable estimates of variable impact with robust standard errors [35-37]. Both analyses using SL and TMLE are conducted in programming language R; the code used to conduct this analysis is publicly available on GitHub (link: https://github.com/ldliao/mexPred).

Results

Descriptive results show the age distribution of laboratory-confirmed patients across the three different epidemic phases **(Supplemental Figure S2**). Phases 1 and 2 have similar distributions, and there are more young people (under 30) in phase 3. The six most prevalent pre-existing conditions are hypertension, obesity, diabetes, smoking, asthma, and renal disease **(Supplemental Figure S3**). The prevalence of all pre-existing conditions decreased over the three phases, and prevalence of hypertension, obesity, and diabetes were drastically reduced in phase 3.

Super learner (SL) prediction

SL fit has high prediction accuracy on the testing set (AUC: 0.907 (95% CI: (0.905-0.908)) (**Table 2**). The SL fit leverages multiple machine learning models: the XGBoost models, generalized additive model, and random forest for prediction (**Supplemental Table S2**). The

For peer review only - http://bmjopen.bmj.com/site/about/guidelines.xhtml

228 simple logistic regression has a lower AUC (testing AUC: 0.874 (95% CI: (0.872-0.876)) than
229 the SL fit, as expected, as it only uses age as a predictor (**Table 2**). However, the simple model is
230 already highly predictive, and the difference is small yet significant. The logistic regression
231 model overpredicts mortality risks for those roughly above age 75 compared to the SL prediction
232 (**Fig. 1**). In line with the simple age-only logistic regression model, permuted variable
233 importance on the SL fit shows, while holding other variables constant, age is consistently the
234 most important for SL prediction in average mortality risk (**Supplemental Figure S4 and Table**
235 **S3**). Having multiple comorbidities can dramatically increase risk for those individuals (**Fig. 2**).

Table 2. Prediction results

	All time (2020/03-2021/11) AUC (95% CI)	Phase 1 (2020/03-2020/10) AUC (95%CI)	Phase 2 (2020/11-2021/03) AUC (95%CI)	Phase 3 (2021/04-2021/11) AUC (95%CI)
Super learner fit	Training: 0.916 (0.915-0.917)<br><br>Testing: 0.907 (0.905-0.908)	Training: 0.887 (0.885-0.888)<br><br>Testing: 0.873 (0.870-0.876)	Training: 0.904 (0.903-0.906)<br><br>Testing: 0.895 (0.892-0.897)	Training: 0.914 (0.913-0.916)<br><br>Testing: 0.906 (0.902-0.909)
Age only logistic regression fit	Training: 0.874 (0.873-0.875)<br><br>Testing: 0.874 (0.872-0.876)	Training: 0.845 (0.843-0.846)<br><br>Testing: 0.846 (0.842-0.850)	Training: 0.868 (0.866-0.870)<br><br>Testing: 0.871 (0.868-0.874)	Training: 0.867 (0.865-0.869)<br><br>Testing: 0.871 (0.866-0.875)

AUC: area under the receiver operating characteristic curve; CI: confidence interval
Training AUC refers to prediction results using only 80% of the data.
Testing AUC refers to prediction results using only 20% left-out of the data (disjoint from training). The testing set is not used for model development.

236

237 **Relative risks of pre-existing conditions**

For peer review only - http://bmjopen.bmj.com/site/about/guidelines.xhtml

To assess the impact of each pre-existing condition, we estimate their respective relative risks (RRs) of mortality, adjusting for demographic variables. We report the estimated RRs in **Table 3**, ordered by impact (most to least) (**Supplemental Figure S5**). The RRs compare the expected risk if all patients have the pre-existing condition (with) versus if all patients do not have the condition (without). The highest impact pre-existing condition is renal disease (RR: 3.783, 95% CI: (3.705, 3.862)); diabetes, obesity, and hypertension also have high impact individually (RR: 1.432-1.847). Minimal differences between the risk estimates are shown for smoking and asthma (RR: 1.049 and 1.037, respectively).

For peer review only

For peer review only - http://bmjopen.bmj.com/site/about/guidelines.xhtml

Table 3. Targeted maximum likelihood estimation relative risk results for each pre-existing condition

	All time (2020/03-2021/11) Relative Risk (95% CI)	Phase 1 (2020/03-2020/10) Relative Risk (95%CI)	Phase 2 (2020/11-2021/03) Relative Risk (95%CI)	Phase 3 (2021/04-2021/11) Relative Risk (95%CI)
Renal disease	3.783 (3.705, 3.862)	2.588 (2.521, 2.657)	2.994 (2.910, 3.080)	6.638 (6.361, 6.927)
Diabetes	1.847 (1.820, 1.875)	1.536 (1.508, 1.566)	1.594 (1.564, 1.625)	2.508 (2.423, 2.596)
Hypertension	1.745 (1.721, 1.770)	1.427 (1.402, 1.452)	1.500 (1.474, 1.527)	2.356 (2.279, 2.436)
Obesity	1.432 (1.417, 1.447)	1.269 (1.249, 1.288)	1.259 (1.239, 1.279)	1.794 (1.750, 1.840)
Smoking	1.049 (1.030, 1.068)	1.001 (0.975, 1.028)	0.992 (0.966, 1.018)	1.158 (1.107, 1.210)
Asthma	1.037 (1.002, 1.073)	0.941 (0.895, 0.989)	0.942 (0.892, 0.995)	1.223 (1.134, 1.319)

CI: confidence interval

The phase analyses indicate pre-existing conditions are especially important in phase 3. Phase 1 and 2 are very similar in terms of both risk prediction and adjusted mortality risk estimates. However, in phase 3, age is less important in prediction (**Supplemental Table S3)** and RRs drastically increase for every comorbidity. The adjusted risks show the decrease for each pre-existing condition in phase 3 (**Supplemental Table S4)**.

For peer review only

For peer review only - http://bmjopen.bmj.com/site/about/guidelines.xhtml

Discussion

Our analysis of (>1.4 million) laboratory-confirmed COVID-19 patients demonstrates that age is by far the most important predictor of average mortality. For those patients with renal disease, diabetes, hypertension, or obesity, having the comorbidity further increases their risk of mortality. A patient with diabetes, hypertension, and obesity is roughly comparable to a patient 20 years older with none of the conditions, based on the predicted mortality (**Fig. 2**). Thus, having a comorbidity increases risk of mortality and should be considered at any age. The reason that comorbidities add little to the predictive power at younger ages is that hypertension and diabetes are age-related and the reported onset is often for those over 30, so the pre-existing conditions are far less prevalent.

Our prediction results using machine learning methods predict better than previous studies, and we demonstrated the feasibility and robustness of using machine learning methods targeted for prediction and variable impact. SL model prediction has an AUC of 0.907, which is higher than any previous Mexican study (AUCs from 0.634 to 0.824) [9, 38]. Although age has been well reported by previous studies as important [6, 38, 39], our analysis is more robust because we do not assume a pre-specified functional relationship between the explanatory variables and the predicted variable, and thereby avoid any arbitrary groupings into age categories. Moreover, since those above age 60 have a higher prevalence of comorbidities, relying on simple logistic regression models can greatly overpredict the average mortality risk for the elder patients. Our study applies TMLE to estimate the adjusted mortality risk ratios for each comorbidity to provide more robust impact estimates that respect time ordering and account for background variables.

For peer review only

For peer review only - http://bmjopen.bmj.com/site/about/guidelines.xhtml

We find consistent results of comorbidities compared to previous studies, and present phase analyses highlighting the changes in relative risks over time. Previous results from logistic regressions indicated odds ratios of 1.458-2.48 for renal disease, 1.237-1.74 for diabetes, 1.173-1.47 for obesity, 1.194-1.315 for hypertension, 0.852-1.02 for smoking, and 0.74-1.420 for asthma [38–40]. Although our analysis is generally consistent with previous findings, our RR estimations have less uncertainty. Renal disease has the greatest impact on mortality, followed by diabetes, hypertension, and obesity; smoking and asthma have negligible impact on mortality risk.

This phase-specific analysis produced a seemingly paradoxical finding. The impact of comorbidities on predicted mortality decreased with time (primarily between the second and third wave), but the RR on mortality dramatically increased for the same conditions **(Supplemental Table S4 and Figure S5)**. The apparent explanation is that mortality risk for people without the comorbidities fell faster than for people with them, increasing the relative risk. The decrease in mortality risk is multifactorial and includes a decrease in susceptibility over time (due to prior infection and vaccination), improved treatment, enhanced healthcare response and opportunity to be admitted to a hospital or ICU, and less virulent viral subtypes. This implies that as herd immunity increases, medical resources should focus even more on protecting vulnerable people at older age and those with comorbidities since they are even more likely to experience severe outcomes compared to those who are younger and/or healthier.

Readers should be cautious about extrapolating our findings to other populations. Although our sample is large and includes patients from all parts of Mexico, most of the patients were IMSS beneficiaries. In order to access IMSS health services, patients require: a) be a formal-sector worker or retired, b) be a direct dependent of such an employee, c) be a bachelor or

For peer review only - http://bmjopen.bmj.com/site/about/guidelines.xhtml

postgraduate student in a public institution, d) voluntarily enroll by paying a fee. Thus, the IMSS population skews toward the upper half of the income distribution. Populations without similar access to health services may have different results. It is also important to consider the potential impact of data quality. Pre-existing conditions were self-reported and likely also inconsistently recorded, perhaps in systematic ways that could have biased the results. For example, if people with severe diabetes were more likely to report diabetes as a pre-existing condition, we may overestimate the impact of diabetes on mortality.

It is also important to consider what predictive variables are included in this model. We sought to predict risk for an individual in the population using their characteristics prior to infection. In other words, what is this person's risk of death from COVID-19 if they were to be infected? The answer to this question best informs the question of who should be prioritized for protection against infection or for early therapeutic interventions following infection. It does not attempt to predict the likely mortality of a patient who presents to the health services with COVID-19 because information about that patient's severity of their COVID-19-related symptoms will represent important additional predictors of their mortality risk.

For peer review only - http://bmjopen.bmj.com/site/about/guidelines.xhtml

Abbreviations

AUC, area under the receiver operating characteristic curve; CI, confidence interval; COPD, chronic obstructive pulmonary disease; COVID-19, coronavirus disease of 2019; IMSS, Mexican Social Security Institute; PCR, polymerase chain reaction; RR, relative risk; SL, super learner; TMLE, targeted maximum likelihood estimation; XGBoost, extreme gradient boosting

Declarations:

Acknowledgements

We thank the staff of C3.ai DTI for their technical support and our colleagues at University of California, Berkeley, the Mexican National Autonomous University, and the Mexican Social Security Institute (IMSS) for all of the administrative and technical support that has allowed this collaboration to flourish.

Authors' contributions

LDL and AEH contributed to the study design and methodology. AJ, YY, and IA contributed to data acquisition. LDL, YY, and KK contributed to data cleaning. LDL led the data analysis and visualization. LDL, AEH, JPG, and SMB interpreted the results. LDL drafted the manuscript with support from RG on literature search. AEH and SMB significantly contributed to the revision of the manuscript. All authors participated in review and edited the manuscript; all authors have read and approved the final manuscript. All authors had full access to all the data in the study and accepted responsibility to submit for publication. All authors take responsibility for the integrity of the data and the accuracy of the data analysis.

For peer review only - http://bmjopen.bmj.com/site/about/guidelines.xhtml

Funding

C3.ai Digital Transformation Institute, Award/Grant no: NA. National Science Foundation, Award/Grant no: DGE 2146752. Bill & Melinda Gates Foundation, Award/Grant no: OPP1165144.

Competing interests

All authors declare no competing interests.

Patient consent for publication

Not required.

Ethics approval

This data-only study was approved on November 4th, 2020, by the Scientific Research National Committee (Mexican Social Security Institute) with R-2020-785-165. The University of California, Berkeley Institutional Review Board (IRB) determined that the project was exempt from IRB approval.

Availability of data and materials

The study was conducted using confidential patient records subject to strict access controls and we are therefore unable to share the data that were used for this study.

Consent for publication

Not applicable.

For peer review only

For peer review only - http://bmjopen.bmj.com/site/about/guidelines.xhtml

References

1. Shang W, Wang Y, Yuan J, *et al*. Global Excess Mortality during COVID-19 Pandemic: A Systematic Review and Meta-Analysis. *Vaccines (Basel)* 2022;**10**. doi:10.3390/vaccines10101702.

2. Hopkins. University of Medicine. Coronavirus Resource Center. *Data Stream* 2020.

3. Singer M. Deadly Companions: COVID-19 and Diabetes in Mexico. *Med Anthropol* 2020;**39**:660–5.

4. Bello-Chavolla OY, Bahena-López JP, Antonio-Villa NE, *et al.* Predicting Mortality Due to SARS-CoV-2: A Mechanistic Score Relating Obesity and Diabetes to COVID-19 Outcomes in Mexico. *J Clin Endocrinol Metab* 2020;**105**. doi:10.1210/clinem/dgaa346

5. Noyola DE, Hermosillo-Arredondo N, Ramírez-Juárez C, *et al.* Association between obesity and diabetes prevalence and COVID-19 mortality in Mexico: an ecological study. *J Infect Dev Ctries* 2021;**15**:1396–403.

6. Giannouchos TV, Sussman RA, Mier Odriozola JM, *et al.* Characteristics and risk factors for COVID-19 diagnosis and adverse outcomes in Mexico: an analysis of 89,756 laboratory–confirmed COVID-19 cases. bioRxiv. 2020. doi:10.1101/2020.06.04.20122481

7. Peña JE la, Rascón-Pacheco RA, Ascencio-Montiel I de J, *et al.* Hypertension, Diabetes and Obesity, Major Risk Factors for Death in Patients with COVID-19 in Mexico. *Arch Med Res* 2021;**52**:443–9.

8. Gutierrez JP, Bertozzi SM. Non-communicable diseases and inequalities increase risk of death among COVID-19 patients in Mexico. *PLoS One* 2020;**15**:e0240394.

9. Martínez-Martínez MU, Alpízar-Rodríguez D, Flores-Ramírez R, *et al.* An Analysis COVID-19 in Mexico: a Prediction of Severity. *J Gen Intern Med* 2022;**37**:624–31.

10. Gimeno-Miguel A, Bliek-Bueno K, Poblador-Plou B, *et al.* Chronic diseases associated with increased likelihood of hospitalization and mortality in 68,913 COVID-19 confirmed cases in Spain: A population-based cohort study. *PLoS One* 2021;**16**:e0259822.

11. Soares R de CM, Mattos LR, Raposo LM. Risk Factors for Hospitalization and Mortality due to COVID-19 in Espírito Santo State, Brazil. *Am J Trop Med Hyg* 2020;**103**:1184–90.

12. Wollenstein-Betech S, Silva AAB, Fleck JL, *et al.* Physiological and socioeconomic characteristics predict COVID-19 mortality and resource utilization in Brazil. *PLoS One* 2020;**15**:e0240346.

13. Atkins JL, Masoli JAH, Delgado J, *et al.* Preexisting Comorbidities Predicting COVID-19 and Mortality in the UK Biobank Community Cohort. *J Gerontol A Biol Sci Med Sci* 2020;**75**:2224–30.

For peer review only - http://bmjopen.bmj.com/site/about/guidelines.xhtml

14. Semenzato, Botton, Drouin, *et al.* Chronic diseases, health conditions and risk of COVID-19-related hospitalization and in-hospital mortality during the first wave of the epidemic in France: a …. *The Lancet Regional* https://www.sciencedirect.com/science/article/pii/S2666776221001356

15. Ge E, Li Y, Wu S, *et al.* Association of pre-existing comorbidities with mortality and disease severity among 167,500 individuals with COVID-19 in Canada: A population-based cohort study. *PLoS One* 2021;**16**:e0258154.

16. Sawadogo W, Tsegaye M, Gizaw A, *et al*. Overweight and obesity as risk factors for COVID-19-associated hospitalisations and death: systematic review and meta-analysis. *BMJ Nutr Prev Health* 2022;**5**:10–8.

17. Kastora S, Patel M, Carter B, *et al*. Impact of diabetes on COVID-19 mortality and hospital outcomes from a global perspective: An umbrella systematic review and meta-analysis. *Endocrinol Diabetes Metab* 2022;**5**:e00338.

18. Akbari A, Fathabadi A, Razmi M, *et al*. Characteristics, risk factors, and outcomes associated with readmission in COVID-19 patients: A systematic review and meta-analysis. *Am J Emerg Med* 2022;**52**:166–73.

19. van der Laan MJ, Polley EC, Hubbard AE. Super learner. *Stat Appl Genet Mol Biol* 2007;**6**:Article25.

20. Polley EC, van der Laan MJ. Super Learner In Prediction. Published Online First: 2010.https://biostats.bepress.com/ucbbiostat/paper266/?TB_iframe=true&width=370.8&height=658.8 (accessed 26 Jul 2022).

21. Petersen ML, van der Laan MJ. Causal models and learning from data: integrating causal modeling and statistical estimation. *Epidemiology* 2014;**25**:418–26.

22. Juárez-Flores A, Ascencio-Montiel IJ, Gutiérrez JP, *et al.* COVID-19 in the Mexican Social Security Institute (IMSS) population. Prevalent symptoms. bioRxiv. 2022. doi:10.1101/2022.04.12.22273734

23. Vigilancia de variantes del virus SARS-CoV-2. Vigilancia de variantes del virus SARS-CoV-2. https://salud.conacyt.mx/coronavirus/variantes/. (accessed 29 Jul 2022).

24. Chipman HA, George EI, McCulloch RE. BART: Bayesian additive regression trees. *aoas* 2010;**4**:266–98.

25. Dey DK, Ghosh SK, Mallick BK. *Generalized linear models: A Bayesian perspective*. CRC Press 2000.

26. Hans C. Elastic Net Regression Modeling With the Orthant Normal Prior. *J Am Stat Assoc* 2011;**106**:1383–93.

For peer review only

For peer review only - http://bmjopen.bmj.com/site/about/guidelines.xhtml

27. Liu. Generalized additive model. *Rep Univ Jyvaskyla Dep Math Stat* Published Online First: 2008.http://people.vcu.edu/~dbandyop/BIOS625/GAM.pdf

28. Tibshirani R. Regression Shrinkage and Selection via the Lasso. *J R Stat Soc Series B Stat Methodol* 1996;**58**:267–88.

29. Friedman JH. Multivariate Adaptive Regression Splines. *aos* 1991;**19**:1–67.

30. Breiman L. Random Forests. *Mach Learn* 2001;**45**:5–32.

31. Hoerl AE, Kennard RW. Ridge Regression: Biased Estimation for Nonorthogonal Problems. *Technometrics* 1970;**12**:55–67.

32. Chen T, Guestrin C. XGBoost: A Scalable Tree Boosting System. In: *Proceedings of the 22nd ACM SIGKDD International Conference on Knowledge Discovery and Data Mining*. New York, NY, USA: : Association for Computing Machinery 2016. 785–94.

33. LeDell E, Petersen M, van der Laan M. Computationally efficient confidence intervals for cross-validated area under the ROC curve estimates. *Electron J Stat* 2015;**9**:1583–607.

34. Rosenbaum PR. Model-Based Direct Adjustment. *J Am Stat Assoc* 1987;**82**:387–94.

35. van der Laan MJ, Rubin D. Targeted Maximum Likelihood Learning. *Int J Biostat* 2006;**2**. doi:10.2202/1557-4679.1043

36. Hubbard AE, Kennedy CJ, van der Laan MJ. Data-Adaptive Target Parameters. In: van der Laan MJ, Rose S, eds. *Targeted Learning in Data Science: Causal Inference for Complex Longitudinal Studies*. Cham: : Springer International Publishing 2018. 125–42.

37. Porter KE, Gruber S, van der Laan MJ, *et al.* The relative performance of targeted maximum likelihood estimators. *Int J Biostat* 2011;**7**. doi:10.2202/1557-4679.1308

38. Wollenstein-Betech S, Cassandras CG, Paschalidis IC. Personalized predictive models for symptomatic COVID-19 patients using basic preconditions: Hospitalizations, mortality, and the need for an ICU or ventilator. *Int J Med Inform* 2020;**142**:104258.

39. Parra-Bracamonte GM, Lopez-Villalobos N, Parra-Bracamonte FE. Clinical characteristics and risk factors for mortality of patients with COVID-19 in a large data set from Mexico. *Ann Epidemiol* 2020;**52**:93–8.e2.

40. Hernández-Galdamez DR, González-Block MÁ, Romo-Dueñas DK, *et al.* Increased Risk of Hospitalization and Death in Patients with COVID-19 and Pre-existing Noncommunicable Diseases and Modifiable Risk Factors in Mexico. *Arch Med Res* 2020;**51**:683–9.

For peer review only - http://bmjopen.bmj.com/site/about/guidelines.xhtml

Fig. 1. Mortality risk prediction comparing age only logistic regression and super learner

GAM: generalized additive model
The smoothed true mortality risk curve is generated using a GAM with integrated smoothness estimation fitted with cubic splines.

For peer review only

For peer review only - http://bmjopen.bmj.com/site/about/guidelines.xhtml

Fig. 2. Super learner predicted mortality risk averaged by specific age in two subgroups: those having all obesity, diabetes, and hypertension pre-existing conditions versus those without

For peer review only

For peer review only - http://bmjopen.bmj.com/site/about/guidelines.xhtml

Supplemental Material

SupplementalMaterial.pdf:

Table S1. TableS1 – [Complete table of baseline variables and pre-existing conditions]

Table S2. TableS2 – [Weighted combination of the super learner fit]

Table S3. TableS3 – [Top 5 ranked most important variables for prediction]

Table S4. TableS4 – [Targeted maximum likelihood estimation adjusted mortality risk, with or without the pre-existing condition]

Figure S1. FigS1 – [Flowchart for analytic sample development]

Figure S2. FigS2 – [Age distribution for laboratory-confirmed COVID-19 patients]

Figure S3. FigS3 – [Prevalence of pre-existing conditions prevalence over time]

Figure S4. FigS4 – [Prediction variable importance predicted using the super learner fit]

Figure S5. FigS5 – [Relative risk for each pre-existing condition associated with mortality]

For peer review only - http://bmjopen.bmj.com/site/about/guidelines.xhtml

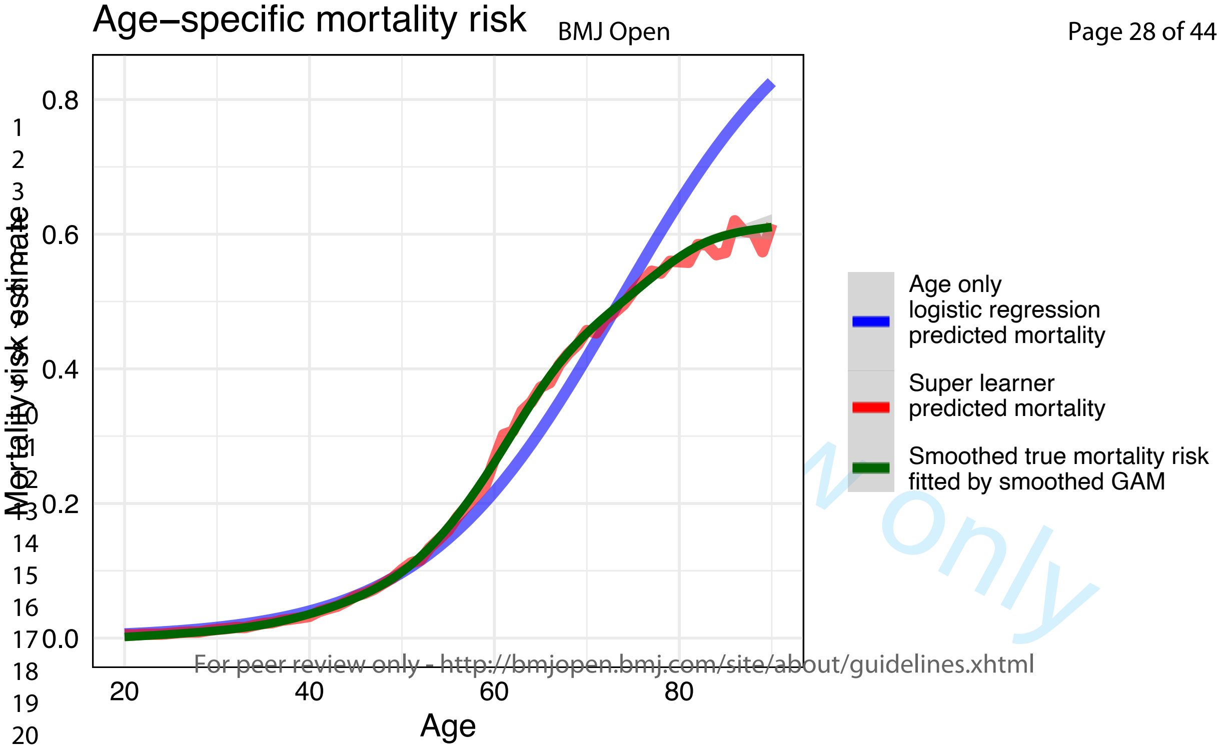

For peer review only - http://bmjopen.bmj.com/site/about/guidelines.xhtml

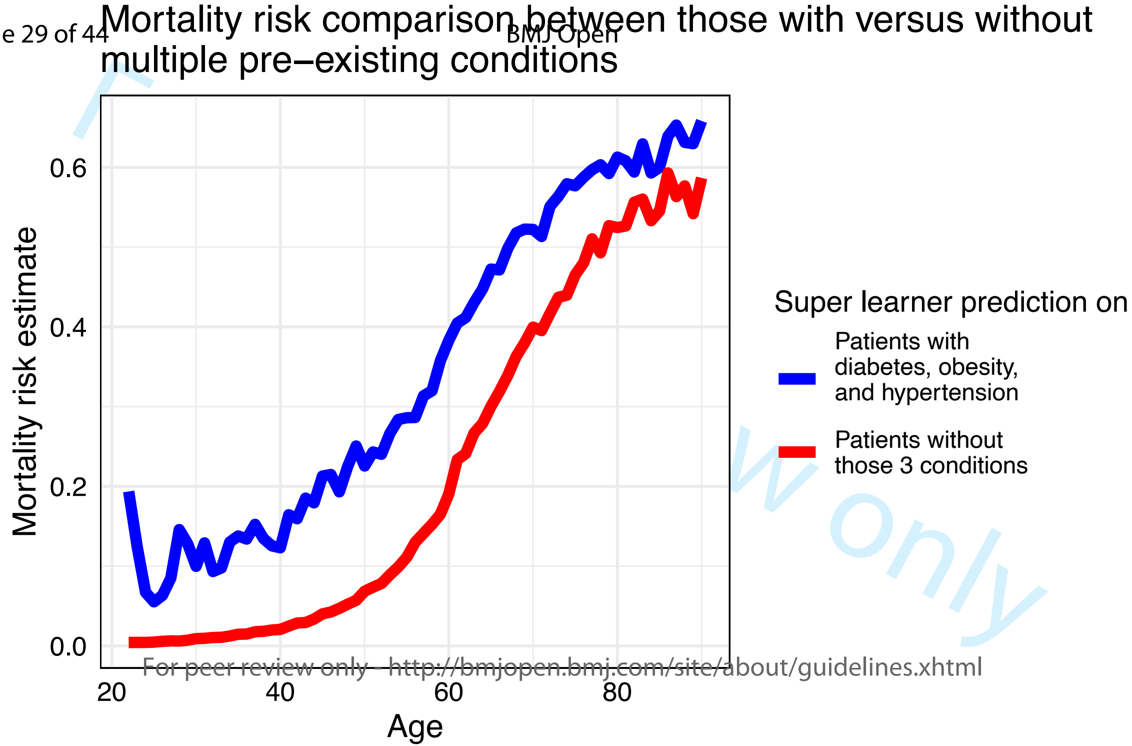
Mortality risk comparison between those with versus without multiple pre–existing conditions
Mortality risk estimate
0.6
0.4
0.2
0.0
20
40
60
80
Age
Super learner prediction on
Patients with diabetes, obesity, and hypertension
Patients without those 3 conditions

Supplemental Material

Who is most at risk of dying if infected with SARS-CoV-2? A mortality risk factor analysis using machine learning of COVID-19 patients over time: a large population-based cohort study in Mexico.

Lauren D. Liao,* Alan E. Hubbard, Juan Pablo Gutiérrez, Arturo Juárez-Flores, Kendall Kikkawa, Ronit Gupta, Yana Yarmolich, Iván de Jesús Ascencio-Montiel, Stefano M. Bertozzi

For peer review only

For peer review only - http://bmjopen.bmj.com/site/about/guidelines.xhtml

Table of Contents

Table S1. Complete table of baseline variables and pre-existing conditions 3

Table S2. Weighted combination of the super learner fit 7

Table S3. Top 5 ranked most important variables for prediction 8

Table S4. Targeted maximum likelihood estimation adjusted mortality risk, with or without the pre-existing condition 9

Fig. S1. Flowchart for analytic sample development 10

Fig. S2. Age distribution for laboratory-confirmed COVID-19 patients 11

Fig. S3. Prevalence of pre-existing conditions prevalence over time 12

Fig. S4. Prediction variable importance predicted using the super learner fit 13

Fig. S5. Relative risk for each pre-existing condition associated with mortality 14

For peer review only

For peer review only - http://bmjopen.bmj.com/site/about/guidelines.xhtml

Table S1. Complete table of baseline variables and pre-existing conditions

	All time (2020/03-2021/11)	Phase 1 (2020/03-2020/10)	Phase 2 (2020/11-2021/03)	Phase 3 (2021/04-2021/11)
sample size	1,423,720	303,278	425,698	694,744
Demographic variables				
Age in years (mean (SD))	42.15 (15.70)	46.41 (16.04)	44.89 (16.27)	38.61 (14.34)
Sex = male (%)	729,782 (51.3)	158,248 (52.2)	218,165 (51.2)	353,369 (50.9)
Insured by IMSS = 1(%)	1,358,440 (95.4)	288,588 (95.2)	402,754 (94.6)	667,098 (96.0)
Indigenous = 1 (%)	7,381 (0.5)	2,200 (0.7)	1,628 (0.4)	3,553 (0.5)
Year-month patient initiated care				
2020/03	1,061 (0.1)	1,061 (0.3)	0 (0.0)	0 (0.0)
2020/04	10,832 (0.8)	10,832 (3.6)	0 (0.0)	0 (0.0)
2020/05	30,720 (2.2)	30,720 (10.1)	0 (0.0)	0 (0.0)
2020/06	51,079 (3.6)	51,079 (16.8)	0 (0.0)	0 (0.0)
2020/07	60,780 (4.3)	60,780 (20.0)	0 (0.0)	0 (0.0)
2020/08	49,618 (3.5)	49,618 (16.4)	0 (0.0)	0 (0.0)
2020/09	44,758 (3.1)	44,758 (14.8)	0 (0.0)	0 (0.0)
2020/10	54,430 (3.8)	54,430 (17.9)	0 (0.0)	0 (0.0)
2020/11	65,437 (4.6)	0 (0.0)	65,437 (15.4)	0 (0.0)
2020/12	93,748 (6.6)	0 (0.0)	93,748 (22.0)	0 (0.0)
2021/01	145,858 (10.2)	0 (0.0)	145,858 (34.3)	0 (0.0)
2021/02	68,421 (4.8)	0 (0.0)	68,421 (16.1)	0 (0.0)
2021/03	52,234 (3.7)	0 (0.0)	52,234 (12.3)	0 (0.0)
2021/04	35,181 (2.5)	0 (0.0)	0 (0.0)	35,181 (5.1)

For peer review only - http://bmjopen.bmj.com/site/about/guidelines.xhtml

2021/05	26,300 (1.8)	0 (0.0)	0 (0.0)	26,300 (3.8)
2021/06	45,986 (3.2)	0 (0.0)	0 (0.0)	45,986 (6.6)
2021/07	170,212 (12.0)	0 (0.0)	0 (0.0)	170,212 (24.5)
2021/08	249,477 (17.5)	0 (0.0)	0 (0.0)	249,477 (35.9)
2021/09	116,569 (8.2)	0 (0.0)	0 (0.0)	116,569 (16.8)
2021/10	48,515 (3.4)	0 (0.0)	0 (0.0)	48,515 (7.0)
2021/11	2,504 (0.2)	0 (0.0)	0 (0.0)	2,504 (0.4)
Mexican states (%)				
Aguascalientes	26,420 (1.9)	6,897 (2.3)	12,350 (2.9)	7,173 (1.0)
Baja California	43,925 (3.1)	13,677 (4.5)	14,188 (3.3)	16,060 (2.3)
Baja California Sur	24,521 (1.7)	4,300 (1.4)	5,423 (1.3)	14,798 (2.1)
Campeche	9,557 (0.7)	1,728 (0.6)	765 (0.2)	7,064 (1.0)
CDMX 1 Noroeste	32,552 (2.3)	5,374 (1.8)	13,174 (3.1)	14,004 (2.0)
CDMX 2 Noreste	54,249 (3.8)	11,370 (3.7)	20,273 (4.8)	22,606 (3.3)
CDMX 3 Suroeste	42,896 (3.0)	9,701 (3.2)	17,588 (4.1)	15,607 (2.2)
CDMX 4 Sureste	62,097 (4.4)	13,248 (4.4)	25,899 (6.1)	22,950 (3.3)
Chiapas	14,826 (1.0)	2,836 (0.9)	1,801 (0.4)	10,189 (1.5)
Chihuahua	23,229 (1.6)	6,489 (2.1)	6,879 (1.6)	9,861 (1.4)
Coahuila	48,933 (3.4)	16,355 (5.4)	15,459 (3.6)	17,119 (2.5)
Colima	18,310 (1.3)	2,997 (1.0)	2,633 (0.6)	12,680 (1.8)
Durango	19,738 (1.4)	6,228 (2.1)	6,674 (1.6)	6,836 (1.0)
Guanajuato	61,570 (4.3)	11,595 (3.8)	29,274 (6.9)	20,701 (3.0)
Guerrero	23,871 (1.7)	4,502 (1.5)	3,920 (0.9)	15,449 (2.2)
Hidalgo	23,673 (1.7)	4,658 (1.5)	8,266 (1.9)	10,749 (1.5)
Jalisco	104,054 (7.3)	19,491 (6.4)	27,868 (6.5)	56,695 (8.2)

For peer review only - http://bmjopen.bmj.com/site/about/guidelines.xhtml

Mexico Oriente	108,067 (7.6)	20,368 (6.7)	39,633 (9.3)	48,066 (6.9)
Mexico Poniente	48,973 (3.4)	11,632 (3.8)	17,026 (4.0)	20,315 (2.9)
Michoacan	30,570 (2.1)	6,246 (2.1)	7,221 (1.7)	17,103 (2.5)
Morelos	18,797 (1.3)	2,845 (0.9)	6,817 (1.6)	9,135 (1.3)
Nayarit	23,934 (1.7)	3,378 (1.1)	2,994 (0.7)	17,562 (2.5)
Nuevo Leon	105,912 (7.4)	23,776 (7.8)	30,114 (7.1)	52,022 (7.5)
Oaxaca	24,324 (1.7)	4,493 (1.5)	5,391 (1.3)	14,440 (2.1)
Puebla	50,998 (3.6)	9,287 (3.1)	17,932 (4.2)	23,779 (3.4)
Queretaro	41,977 (2.9)	4,707 (1.6)	19,259 (4.5)	18,011 (2.6)
Quintana Roo	38,390 (2.7)	4,607 (1.5)	4,542 (1.1)	29,241 (4.2)
San Luis Potosi	26,118 (1.8)	7,353 (2.4)	7,573 (1.8)	11,192 (1.6)
Sinaloa	44,333 (3.1)	11,030 (3.6)	9,405 (2.2)	23,898 (3.4)
Sonora	27,691 (1.9)	7,245 (2.4)	6,083 (1.4)	14,363 (2.1)
Tabasco	16,004 (1.1)	2,622 (0.9)	1,719 (0.4)	11,663 (1.7)
Tamaulipas	38,941 (2.7)	8,504 (2.8)	7,442 (1.7)	22,995 (3.3)
Tlaxcala	13,809 (1.0)	2,769 (0.9)	4,957 (1.2)	6,083 (0.9)
Veracruz Norte	41,804 (2.9)	10,002 (3.3)	6,801 (1.6)	25,001 (3.6)
Veracruz Sur	35,019 (2.5)	10,292 (3.4)	4,970 (1.2)	19,757 (2.8)
Yucatan	35,126 (2.5)	5,968 (2.0)	5,091 (1.2)	24,067 (3.5)
Zacatecas	18,512 (1.3)	4,708 (1.6)	8,294 (1.9)	5,510 (0.8)
Pre-existing conditions				
Asthma = yes (%)	25,297 (1.8)	7,951 (2.6)	7,765 (1.8)	9,581 (1.4)
Cardiovascular disease = yes (%)	17,816 (1.3)	6,643 (2.2)	6,389 (1.5)	4,784 (0.7)
Chronic liver disease = yes (%)	1,875 (0.1)	710 (0.2)	668 (0.2)	497 (0.1)
COPD = yes (%)	15,390 (1.1)	5,825 (1.9)	5,496 (1.3)	4,069 (0.6)

For peer review only

For peer review only - http://bmjopen.bmj.com/site/about/guidelines.xhtml

Diabetes = yes (%)	169,869 (11.9)	55,551 (18.3)	61,120 (14.4)	53,198 (7.7)
Hemolytic anemia = yes (%)	705 (0.0)	276 (0.1)	246 (0.1)	183 (0.0)
HIV = yes (%)	4,717 (0.3)	1,133 (0.4)	1,425 (0.3)	2,159 (0.3)
Hypertension = yes (%)	228,901 (16.1)	72,615 (23.9)	83,735 (19.7)	72,551 (10.4)
Immunosuppression = yes (%)	10,434 (0.7)	4,102 (1.4)	3,453 (0.8)	2,879 (0.4)
Neurological disease = yes (%)	1,645 (0.1)	544 (0.2)	559 (0.1)	542 (0.1)
Obesity = yes (%)	181,736 (12.8)	55,965 (18.5)	60,217 (14.1)	65,554 (9.4)
Smoking = yes (%)	87,161 (6.1)	21,253 (7.0)	28,346 (6.7)	37,562 (5.4)
Cancer diagnosis = yes (%)	3,751 (0.3)	1,178 (0.4)	1,317 (0.3)	1,256 (0.2)
Renal disease diagnosis = yes (%)	24,099 (1.7)	8,912 (2.9)	8,555 (2.0)	6,632 (1.0)
Tuberculosis = yes (%)	675 (0.0)	203 (0.1)	218 (0.1)	254 (0.0)

CDMX: Ciudad de México; COPD: Chronic obstructive pulmonary disease; HIV: human immunodeficiency virus; SD: standard deviation
Mexican states refer to where the patient was treated

For peer review only

Table S2. Weighted combination of the super learner fit

Machine learning candidate algorithm	Weights	Mean squared error	Standard error
Bayesian additive regression trees	0	0.266	0.0002
Bayesian generalized linear model	0	0.067	0.0003
Elastic net regression	0	0.068	0.0004
Empirical mean	0	0.094	0.0005
XGBoost (multiple tuning)	0.596 (combined)	0.065 (on average)	0.0003 (on average)
Generalized additive model	0.222	0.066	0.0004
LASSO regression	0	0.067	0.0004
Logistic regression	0	0.067	0.0004
Multivariate Adaptive Regression Splines	0	0.068	0.0004
Random forest	0.181	0.066	0.0002
Ridge regression	0	0.067	0.0002

LASSO: least absolute shrinkage and selection operator; XGBoost: extreme gradient boosting
XGBoost coefficients are combined; mean squared error and standard error were averaged.

For peer review only - http://bmjopen.bmj.com/site/about/guidelines.xhtml

Table S3. Top 5 ranked most important variables for prediction

	All time (2020/03-2021/11)	Phase 1 (2020/03-2020/10)	Phase 2 (2020/11-2021/03)	Phase 3 (2021/04-2021/11)
Rank 1	Age 0.147	Age 0.209	Age 0.208	Age 0.069
Rank 2	Year-month patient initiated care 0.014	Renal disease 0.008	Mexican state 0.007	Renal disease 0.004
Rank 3	Renal disease 0.005	Sex 0.007	Renal disease 0.006	Mexican state 0.003
Rank 4	Mexican state 0.004	Year-month patient initiated care 0.007	Insured by IMSS 0.006	Diabetes 0.003
Rank 5	Sex 0.004	Mexican state 0.006	Sex 0.006	Insured by IMSS 0.002

IMSS: Mexican Institute of Social Security
Mexican state refers to where the patient was treated.
Measured by the log-likelihood difference in prediction pre-post permutation of each variable while holding all others constant.

For peer review only

Table S4. Targeted maximum likelihood estimation adjusted mortality risk, with or without the pre-existing condition

	All time (2020/03-2021/11)		Phase 1 (2020/03-2020/10)		Phase 2 (2020/11-2021/03)		Phase 3 (2021/04-2021/11)	
	with	without	with	without	with	without	with	without
Renal disease	0.381	0.101	0.439	0.170	0.425	0.142	0.305	0.046
Diabetes	0.173	0.094	0.247	0.161	0.214	0.135	0.104	0.041
Hypertension	0.162	0.093	0.231	0.162	0.201	0.134	0.097	0.041
Obesity	0.141	0.099	0.212	0.168	0.177	0.141	0.080	0.045
Smoking	0.110	0.105	0.176	0.176	0.146	0.147	0.056	0.048
Asthma	0.109	0.105	0.166	0.177	0.139	0.147	0.059	0.049

For peer review only

Fig. S1. Flowchart for analytic sample development

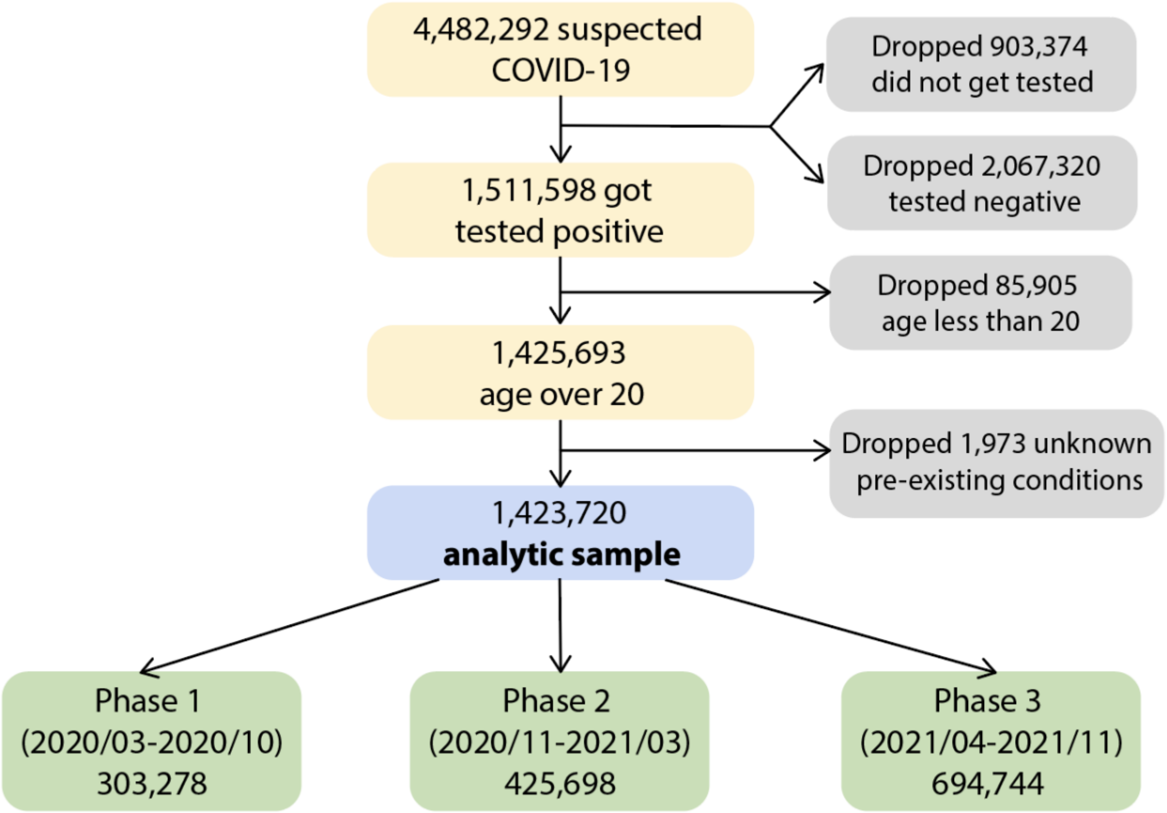

For peer review only - http://bmjopen.bmj.com/site/about/guidelines.xhtml

Fig. S2. Age distribution for laboratory-confirmed COVID-19 patients

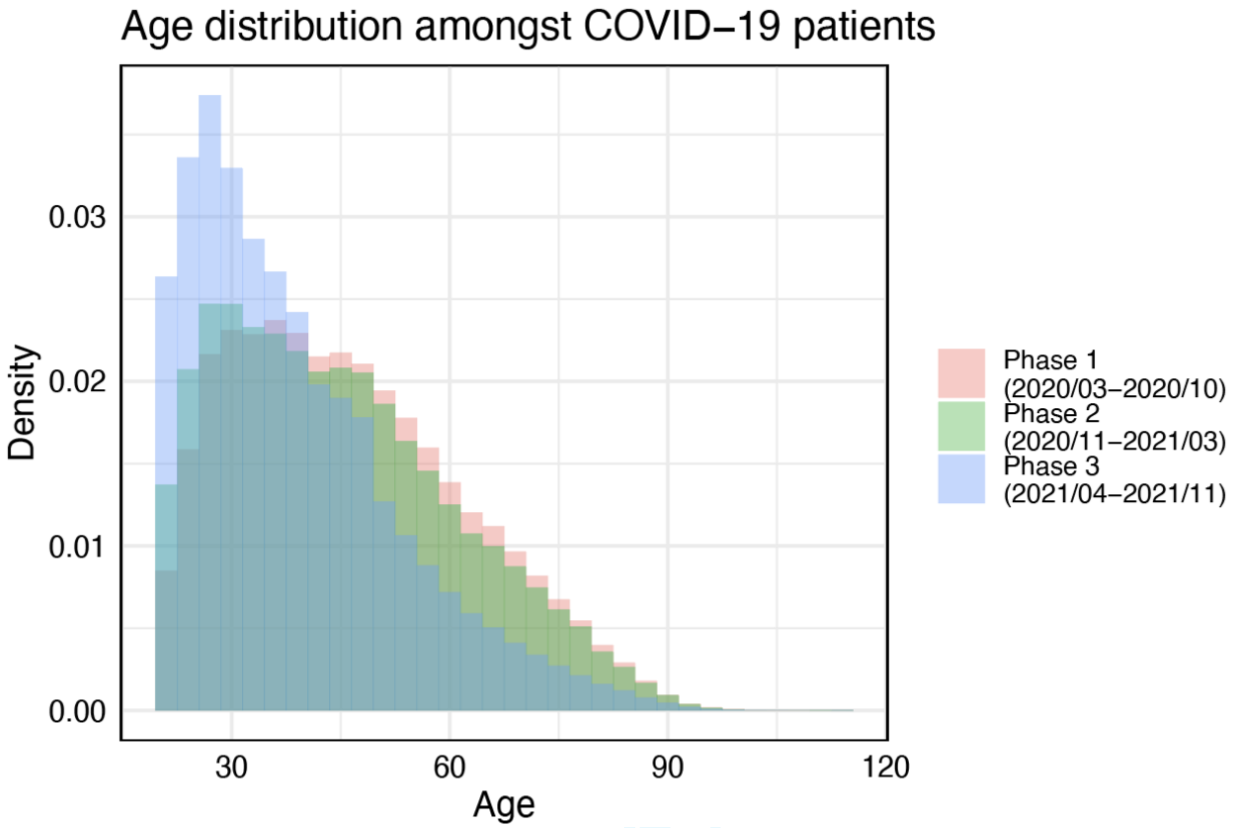

For peer review only - http://bmjopen.bmj.com/site/about/guidelines.xhtml

Fig. S3. Prevalence of pre-existing conditions prevalence over time

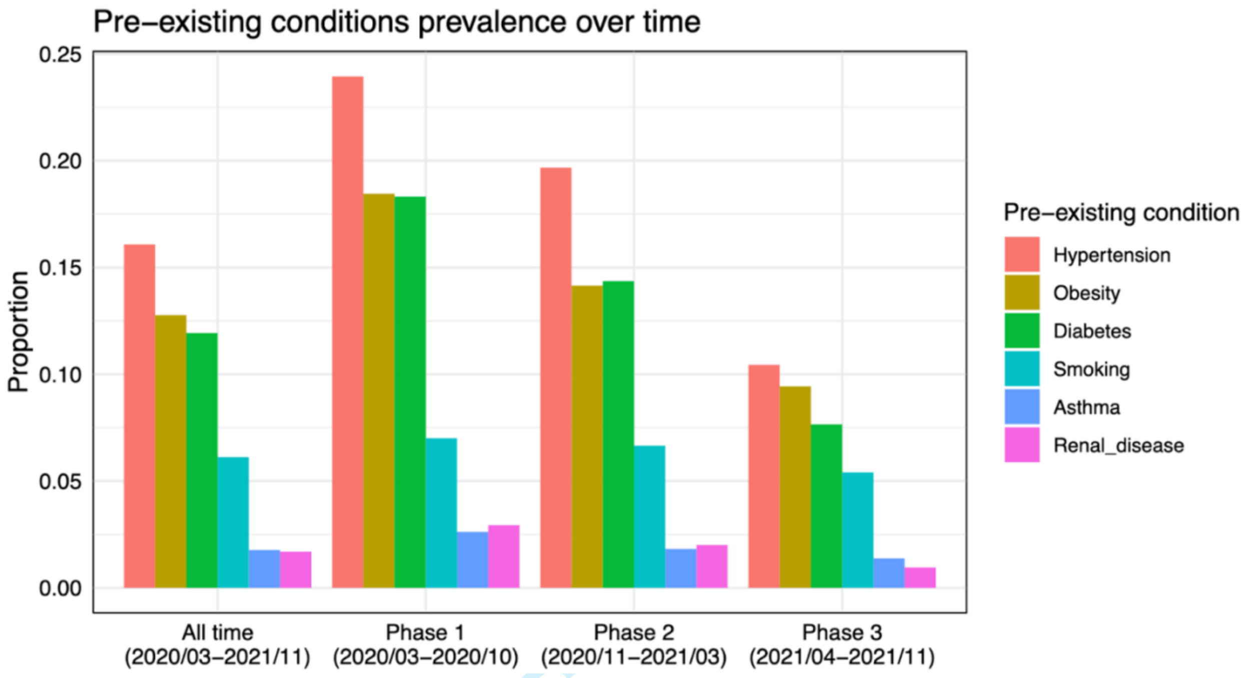

For peer review only - http://bmjopen.bmj.com/site/about/guidelines.xhtml

Fig. S4. Prediction variable importance predicted using the super learner fit

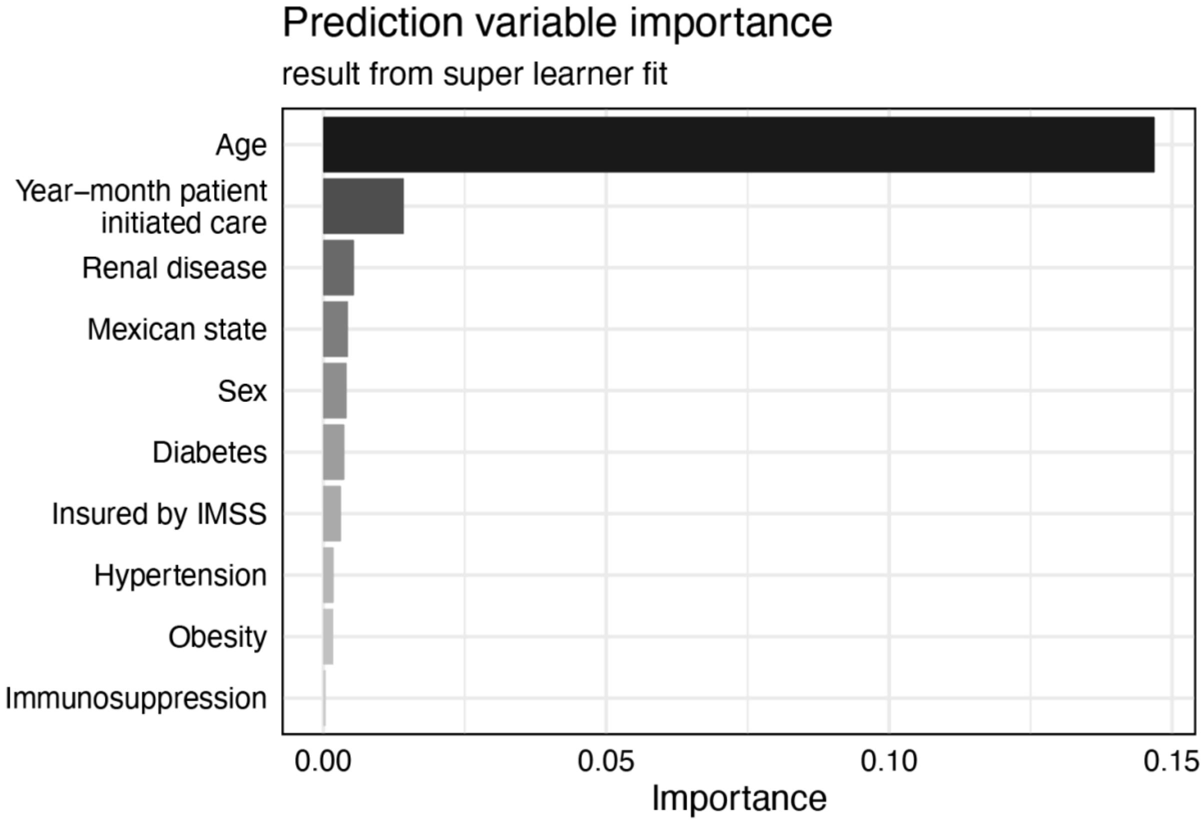

IMSS: Mexican Institute of Social Security

For peer review only - http://bmjopen.bmj.com/site/about/guidelines.xhtml

Fig. S5. Relative risk for each pre-existing condition associated with mortality

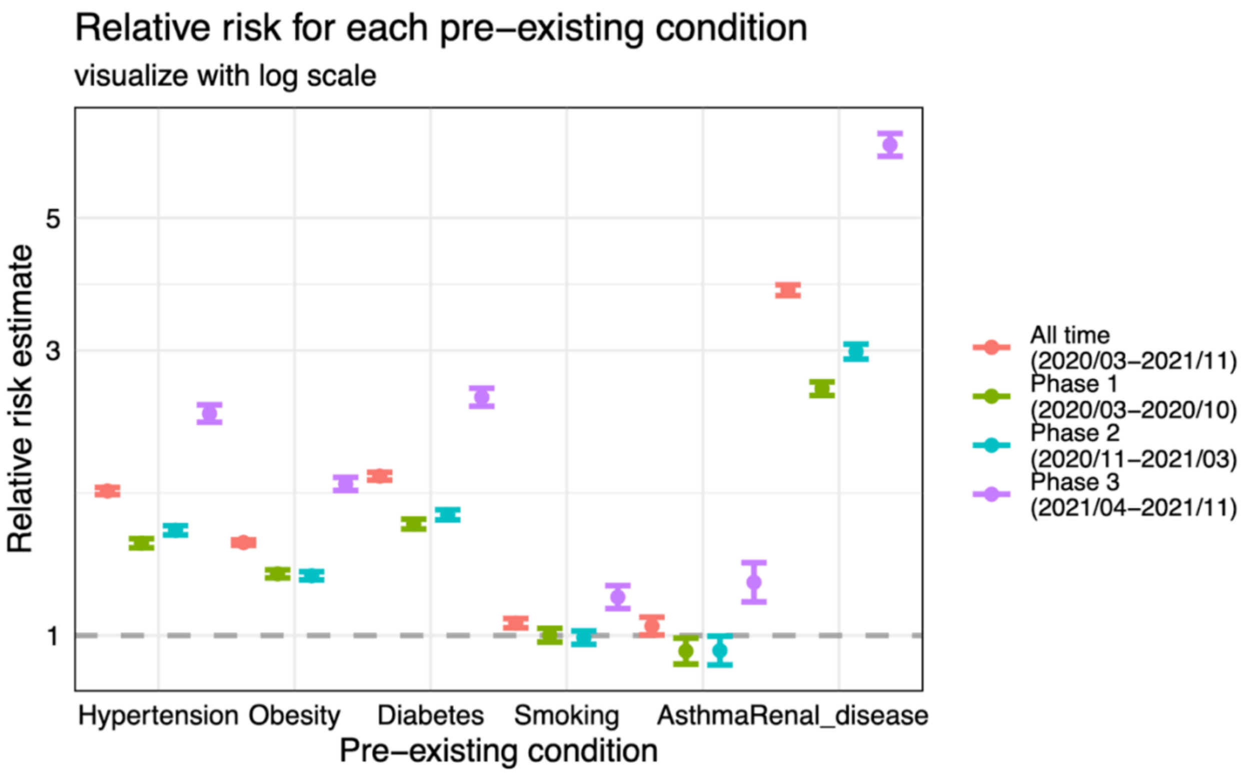

Error bars reflect a 95% confidence interval.

For peer review only - http://bmjopen.bmj.com/site/about/guidelines.xhtml

STROBE Statement—Checklist of items that should be included in reports of ***cohort studies***

	Item No	Recommendation	Page No
**Title and abstract**	1	(*a*) Indicate the study's design with a commonly used term in the title or the abstract	1
		(*b*) Provide in the abstract an informative and balanced summary of what was done and what was found	2-3
**Introduction**			
Background/rationale	2	Explain the scientific background and rationale for the investigation being reported	5-7
Objectives	3	State specific objectives, including any prespecified hypotheses	7
**Methods**			
Study design	4	Present key elements of study design early in the paper	7-8
Setting	5	Describe the setting, locations, and relevant dates, including periods of recruitment, exposure, follow-up, and data collection	7-8
Participants	6	(*a*) Give the eligibility criteria, and the sources and methods of selection of participants. Describe methods of follow-up	7-8
		(*b*) For matched studies, give matching criteria and number of exposed and unexposed	N/A
Variables	7	Clearly define all outcomes, exposures, predictors, potential confounders, and effect modifiers. Give diagnostic criteria, if applicable	8-10
Data sources/ measurement	8*	For each variable of interest, give sources of data and details of methods of assessment (measurement). Describe comparability of assessment methods if there is more than one group	8
Bias	9	Describe any efforts to address potential sources of bias	16
Study size	10	Explain how the study size was arrived at	9-10
Quantitative variables	11	Explain how quantitative variables were handled in the analyses. If applicable, describe which groupings were chosen and why	8-9
Statistical methods	12	(*a*) Describe all statistical methods, including those used to control for confounding	10-12
		(*b*) Describe any methods used to examine subgroups and interactions	9-10
		(*c*) Explain how missing data were addressed	9
		(*d*) If applicable, explain how loss to follow-up was addressed	N/A
		(*e*) Describe any sensitivity analyses	N/A
**Results**			
Participants	13*	(a) Report numbers of individuals at each stage of study—eg numbers potentially eligible, examined for eligibility, confirmed eligible, included in the study, completing follow-up, and analysed	9-10
		(b) Give reasons for non-participation at each stage	9
		(c) Consider use of a flow diagram	9
Descriptive data	14*	(a) Give characteristics of study participants (eg demographic, clinical, social) and information on exposures and potential confounders	8-9
		(b) Indicate number of participants with missing data for each variable of interest	N/A
		(c) Summarise follow-up time (eg, average and total amount)	8-9
Outcome data	15*	Report numbers of outcome events or summary measures over time	9

For peer review only - http://bmjopen.bmj.com/site/about/guidelines.xhtml

Main results	16	(*a*) Give unadjusted estimates and, if applicable, confounder-adjusted estimates and their precision (eg, 95% confidence interval). Make clear which confounders were adjusted for and why they were included	13
		(*b*) Report category boundaries when continuous variables were categorized	N/A
		(*c*) If relevant, consider translating estimates of relative risk into absolute risk for a meaningful time period	14
Other analyses	17	Report other analyses done—eg analyses of subgroups and interactions, and sensitivity analyses	13-14
**Discussion**			
Key results	18	Summarise key results with reference to study objectives	15
Limitations	19	Discuss limitations of the study, taking into account sources of potential bias or imprecision. Discuss both direction and magnitude of any potential bias	16-17
Interpretation	20	Give a cautious overall interpretation of results considering objectives, limitations, multiplicity of analyses, results from similar studies, and other relevant evidence	16
Generalisability	21	Discuss the generalisability (external validity) of the study results	16-17
**Other information**			
Funding	22	Give the source of funding and the role of the funders for the present study and, if applicable, for the original study on which the present article is based	19

*Give information separately for exposed and unexposed groups.

Note: An Explanation and Elaboration article discusses each checklist item and gives methodological background and published examples of transparent reporting. The STROBE checklist is best used in conjunction with this article (freely available on the Web sites of PLoS Medicine at http://www.plosmedicine.org/, Annals of Internal Medicine at http://www.annals.org/, and Epidemiology at http://www.epidem.com/). Information on the STROBE Initiative is available at http://www.strobe-statement.org.

For peer review only
